# Supplementary material for: Product innovation design process combined Kano and TRIZ with AD: Case study
Source: PLoS One. 2024 Mar 28;19(3):e0296980. doi: 10.1371/journal.pone.0296980 (PMC10977887; doi:10.1371/journal.pone.0296980)
Supplement: S1 File — (PDF) [file pone.0296980.s001.pdf]

Original Data of Kano Questionnaire on Research of Self-balancing Two-wheeled Vehicle Functionality

Question 1: Commuting Functionality (Ease of standing on the vehicle)

Question \ Options	Excellent	Good	Average	Fair	Poor
If it is convenient for the rider to stand on the self-balancing two-wheeled vehicle, what is your evaluation?	5(11.9%)	34(80.95%)	2(4.76%)	1(2.38%)	0(0%)
If it is not convenient for the rider to stand on the self-balancing two-wheeled vehicle, what is your evaluation?	0(0%)	1(2.38%)	3(7.14%)	1(2.38%)	37(88.1%)
Summary	5(5.95%)	35(41.67%)	5(5.95%)	2(2.38%)	37(44.05%)

Question 2: Commuting Functionality (Riding speed)

Question \ Options	Excellent	Good	Average	Fair	Poor
If the self-balancing two-wheeled vehicle has a fast riding speed, what is your evaluation?	15(35.71%)	21(50%)	3(7.14%)	3(7.14%)	0(0%)
If the self-balancing two-wheeled vehicle has a low riding speed, what is your evaluation?	0(0%)	0(0%)	5(11.9%)	26(61.9%)	11(26.19%)
Summary	15(17.86%)	21(25%)	8(9.52%)	29(34.52%)	11(13.1%)

Question 3: Safety (Stable structures)

Question \ Options	Excellent	Good	Average	Fair	Poor
If the body structure of the self-balancing two-wheeled vehicle is stable, what is your evaluation?	5(11.9%)	37(88.1%)	0(0%)	0(0%)	0(0%)
If the body structure of the self-balancing two-wheeled vehicle is unstable, what is your evaluation?	0(0%)	0(0%)	0(0%)	2(4.76%)	40(95.24%)
Summary	5(5.95%)	37(44.05%)	0(0%)	2(2.38%)	40(47.62%)

Question 4: Safety (Responsive braking system)

Question \ Options	Excellent	Good	Average	Fair	Poor
If the braking system of the self-balancing two-wheeled vehicle is sensitive, what is your evaluation?	7(16.67%)	35(83.33%)	0(0%)	0(0%)	0(0%)
If the braking system of the self-balancing two-wheeled vehicle is not sensitive, what is your evaluation?	0(0%)	0(0%)	0(0%)	8(19.05%)	34(80.95%)
Summary	7(8.33%)	35(41.67%)	0(0%)	8(9.52%)	34(40.48%)

Question 5: Comfort (Good shock absorption)

Question \ Options	Excellent	Good	Average	Fair	Poor
If the self-balancing two-wheeled vehicle has good shock absorption, what is your evaluation?	35(83.33%)	6(14.29%)	1(2.38%)	0(0%)	0(0%)
If the self-balancing two-wheeled vehicle has poor shock absorption, what is your evaluation?	0(0%)	0(0%)	2(4.76%)	23(54.76%)	17(40.48%)
Summary	35(41.67%)	6(7.14%)	3(3.57%)	23(27.38%)	17(20.24%)

Question 6: Portability (Lightweight)

Question \ Options	Excellent	Good	Average	Fair	Poor
If the body weight of the self-balancing two-wheeled vehicle is light, what is your evaluation?	34(80.95%)	7(16.67%)	1(2.38%)	0(0%)	0(0%)
If the body weight of the self-balancing two-wheeled vehicle is heavy, what is your evaluation?	0(0%)	1(2.38%)	5(11.9%)	28(66.67%)	8(19.05%)
Summary	34(40.48%)	8(9.52%)	6(7.14%)	28(33.33%)	8(9.52%)

Question 7: Portability (Handle for carrying)

Question \ Options	Excellent	Good	Average	Fair	Poor
If the self-balancing two-wheeled vehicle has a handle, what is your evaluation?	26(61.9%)	13(30.95%)	3(7.14%)	0(0%)	0(0%)
If the self-balancing two-wheeled vehicle does not have a handle, what is your evaluation?	0(0%)	0(0%)	18(42.86%)	19(45.24%)	5(11.9%)
Summary	26(30.95%)	13(15.48%)	21(25%)	19(22.62%)	5(5.95%)

Question 8: Portability (Reduced size when idle)

Question \ Options	Excellent	Good	Average	Fair	Poor
If the self-balancing two-wheeled vehicle can reduce its size when not in use, what is your evaluation?	35(83.33%)	4(9.52%)	3(7.14%)	0(0%)	0(0%)
If the self-balancing two-wheeled vehicle cannot reduce its size when not in use, what is your evaluation?	0(0%)	0(0%)	17(40.48%)	22(52.38%)	3(7.14%)
Summary	35(41.67%)	4(4.76%)	20(23.81%)	22(26.19%)	3(3.57%)

Question 9: Maintainability (Simple structures for easy maintenance)

Question \ Options	Excellent	Good	Average	Fair	Poor
If the structure of the self-balancing two-wheeled vehicle is simple, making it easy to repair and replace parts, what is your evaluation?	19(45.24%)	21(50%)	2(4.76%)	0(0%)	0(0%)
If the structure of the self-balancing two-wheeled vehicle is complex, making it easy to repair and replace parts, what is your evaluation?	0(0%)	0(0%)	5(11.9%)	13(30.95%)	24(57.14%)
Summary	19(22.62%)	21(25%)	7(8.33%)	13(15.48%)	24(28.57%)

Question 10: Aesthetics (Aesthetically pleasing appearance)

Question \ Options	Excellent	Good	Average	Fair	Poor
If the appearance of the self-balancing two-wheeled vehicle meets aesthetic requirements, what is your evaluation?	27(64.29%)	13(30.95%)	2(4.76%)	0(0%)	0(0%)
If the appearance of the self-balancing two-wheeled vehicle does not meet aesthetic requirements, what is your evaluation?	0(0%)	0(0%)	7(16.67%)	10(23.81%)	25(59.52%)
Summary	27(32.14%)	13(15.48%)	9(10.71%)	10(11.9%)	25(29.76%)
