# Supplementary material for: Product innovation design process combined Kano and TRIZ with AD: Case study
Source: PLoS One. 2024 Mar 28;19(3):e0296980. doi: 10.1371/journal.pone.0296980 (PMC10977887; doi:10.1371/journal.pone.0296980)
Supplement: Attachment — Submitted filename: Response to Reviewers.docx [file pone.0296980.s002.docx]

Dear reviewers,

Thank you very much for your comments and professional advice on our manuscript entitled “Product innovation design process combined Kano and TRIZ with AD: case study” (Manuscript Number: PONE-D-23-21362). These opinions help to improve the academic quality of our paper. Based on your suggestions and requests, we have modified the original manuscript. The following is a point-by-point response to the questions and comments raised by Reviewer 1 and Reviewer 2.

It should be noted that, due to the length of the response, the answer is divided into two parts. **Part Ⅰ** encompasses Page **2** to Page **9**, which includes responses to Reviewer 1 and relevant references. **Part Ⅱ** covers Page **10** to Page **41**, which includes answers to Reviewer 2 and relevant references.

If any further changes are required, we kindly request you to provide suggestions, and we will make every effort to address them. Finally, we would like to express our sincere gratitude for your time and effort in reviewing the paper.

Thank you and best regards.

Yours sincerely,

Professor Wei Liu

Nanjing Forestry University

159 Longpan Road, Xuanwu District, Nanjing, Jiangsu, China

E-mail: liuwei@njfu.edu.cn

**Part Ⅰ**

Part Ⅰ encompasses Page **2** to Page **9**, which includes responses to Reviewer 1 and relevant references.

The response consists of two parts: the original questions raised by Reviewer 1 and the corresponding answers provided by the authors. To better differentiate the text content, the original questions will be in italics, while the authors' answers will not. The references used during the answering process will be presented upon completion of all question responses.

**Response 1:**

**The original question:**

*How do you ensure that the integration of Axiomatic Design, Kano, and TRIZ is seamless and that the strengths of each theory are effectively leveraged without creating conflicts or redundancies?*

**The authors’ answer:**

The answer will be divided into two parts.

(1) We consider that the integration of Axiomatic Design, Kano, and TRIZ is seamless. The literature review shows that the content generated by these three methods can be utilized as a prerequisite for applying another theory method. Kano aids in researching user requirements [1]. The analysis of user requirements is mapped to functional requirements within the Axiomatic Design (AD) theory, and design contradictions are analyzed with the assistance of the Independence Axiom [2-4]. TRIZ proposes solutions based on the identified contradictions [5]. Lastly, Kano's analyzed user requirements are transformed into evaluation criteria, and with the aid of the Information Axiom in AD theory, design solutions are evaluated [6]. The content generated by these methods during the utilization process can thus be utilized as input when employing another theory method. Therefore, we believe that the integration of these three methods is seamless.

(2) The literature review found that the functional roles of Kano, Axiomatic Design, and TRIZ theories are consistent with the process of product innovation design without generating additional content or adding new contradictions.

First, the process of product innovation design is outlined. Services and products start with analyzing user requirements [7]. User requirements determine the product's functional attributes, while the functionality's realization depends on technical principles and product structures [8]. Analyzing the mapping relationship between functionality and structure and resolving contradictions between functionality and structure are the main tasks of product function design [9]. After completing the design solution, evaluating the design solution will aid designers in making design decisions [10]. In summary, the complete process of product innovation design includes five main steps: user requirement research (S1), requirement-function transformation (S2), analysis of design problems (S3), resolution of design problems (S4), and design solution evaluation (S5).

The following explains the functional characteristics of these three theoretical methods. Kano is a method adept at analyzing user requirements (S1). The mapping of the domains in Axiomatic Design theory can transform user requirements into functional requirements (S2). The independent axioms in Axiomatic Design theory excel at analyzing contradictions between functionality and structure in the design (S3). TRIZ is characterized by providing innovative methods to address design contradictions (S4). The information axiom in Axiomatic Design theory is helpful in evaluating solutions by calculating the amount of information (S5). It should be noted that although Axiomatic Design theory introduces the concept of user domain, it does not provide specific methods for analyzing user requirements. Additionally, although Axiomatic Design theory can explore design contradictions, it does not provide methods for solving them. In conclusion, the integrated new process formed by these three theoretical methods is consistent with the process of product innovation design. Furthermore, each theoretical approach complements the shortcomings of others in functionality without generating contradictions or redundancies.

**Response 2:**

**The original question:**

*Could there be challenges in accurately classifying and prioritizing user needs using the Kano model? How do you address potential variations in user needs within different market segments?*

**The authors’ answer:**

There are indeed challenges in accurately classifying and prioritizing user needs using the Kano model. User needs are dynamic and may vary across different market segments. We believe these are the primary reasons for the challenges that exist.

To address the potential changes in user needs within market segments, we believe two ways can assist. Firstly, we suggest applying the Kano model in agile development processes. An iterative design approach can capture dynamic changes in user needs within market segments [11]. Secondly, combining extensive data analysis and machine learning techniques can allow for more precise analysis and prediction of user needs using user behavior data and feedback data [7,12].

**Response 3:**

**The original question:**

*While the method aims to enhance innovation capability, could the incorporation of TRIZ for solving problems potentially lead to extended design iteration cycles? How can you balance rapid iteration with the thoroughness of TRIZ analysis?*

**The authors’ answer:**

Utilizing TRIZ for problem-solving may lead to an extended design iteration cycle. TRIZ is a complex methodology requiring significant investment in in-depth problem analysis and innovative solution exploration. Additionally, designers still need to rely on their experience to select engineering parameters and inventive principles offered by TRIZ [13]. Multiple trial and error attempts may be necessary to find the correct direction.

We believe that optimizing design management and methodologies can assist in keeping a balance between iterative design and thorough TRIZ analysis. Firstly, parallel TRIZ analysis can be conducted alongside design iterations, allowing for simultaneous rapid iterations and TRIZ analysis, reducing the overall design iteration cycle [14]. Furthermore, a model can be trained using machine learning techniques to assist in selecting engineering parameters and inventive principles [15]. The model reduces the number of trial and error attempts, enabling the rapid identification of innovative solutions.

**Response 4:**

**The original question:**

*Given that this integrated approach requires familiarity with multiple theories, how will designers be trained to effectively use and integrate Axiomatic Design, Kano, and TRIZ? How do you manage the potential learning curve for design teams?*

**The authors’ answer:**

The shape and speed of the potential learning curve depend on various factors, such as learners' background knowledge, learning methods and strategies, learning resources, and environment. Through proper training and guidance, as well as practical application and reflection, learners can effectively cope with challenges in the learning process and accelerate the upward trajectory of the learning curve. Therefore, we propose the following training plan to effectively utilize and integrate Axiomatic Design, Kano, and TRIZ for designers.

Firstly, establish a training program that clearly defines training objectives and determines the sequence of topics to be taught. Based on the design team's needs and project requirements, combine theoretical training with practical examples of Axiomatic Design, Kano, and TRIZ, and provide relevant teaching materials and reference materials. Secondly, organize regular group learning and team discussions, leveraging internal knowledge sharing and experience transfer within the design team to enhance the effectiveness of theoretical learning. Additionally, through project practice, integrate theory with practical application, further familiarizing participants with methodology usage and utilizing real-life cases for feedback and improvement. Finally, encourage the learning team to actively participate in relevant workshops and seminars, actively exploring new approaches and techniques for methodology usage.

**Response 5:**

**The original question:**

*The method is proposed as applicable to various industries. How do you account for the differing nature of products, markets, and user needs across these diverse sectors?*

**The authors’ answer:**

First, the Kano model focuses on classifying and prioritizing user needs. Different industries have unique characteristics in terms of user requirements. For example, in the electronics industry, users may prioritize functionality and performance, while taste and quality may be of more significant concern in the food industry. Therefore, when applying the Kano model, it is necessary to consider the specific characteristics of user needs in different industries and determine the focus of product development accordingly.

Second, Axiomatic Design is a methodology used to analyze contradictions between functions and structures and evaluate design solutions. In the design process, products require different parts and systems based on the demands of distinct industries. For instance, in the automotive industry, designers need to propose different body structures based on the various functions of vehicles. After presenting design solutions, since the emphasis on functions differs across industries, the criteria for design evaluation also vary. For example, medical products emphasize equipment reliability, while children's toys prioritize playfulness. Therefore, when applying Axiomatic Design, it is essential to analyze product structures based on the functional requirements of different industries and establish appropriate design evaluation criteria.

Finally, TRIZ is a methodology for solving technical problems and fostering innovation. The technical issues and innovation challenges faced by different industries also vary. For instance, the manufacturing industry may encounter problems with production efficiency and cost control, while the software development industry may demand feature expansion and user experience. When applying TRIZ, it is necessary to select appropriate problem-solving methods and innovation strategies under the characteristics and requirements of different industries.

Therefore, although methods and tools such as Kano, Axiomatic Design, and TRIZ are applicable across various industries, their practical implementation needs to consider the different nature of products, markets, and user requirements in each industry and accordingly adjust and customize the specific application of these methods.

**Response 6:**

**The original question:**

*How do you balance the potential benefits of the integrated approach with the associated resource costs?*

**The authors’ answer:**

First, it is necessary to conduct an assessment of the advantages of each method, understanding their strengths in problem-solving, improving product quality, and enhancing innovation. Comparisons should be made regarding their applicability and effectiveness in different situations. Second, an evaluation of the resource costs of each method is needed. The resource costs should be considered from the perspectives of learning, time, human resources, technical support, and other factors associated with implementing the methods. Based on the assessments of advantages and resource costs, a selective integration of different theoretical approaches should be carried out according to the nature and requirements of the actual problem. Lastly, continuous assessment of the achieved benefits and costs should be conducted after implementing the integrated method. Based on the real situation, timely adjustments and optimizations of the method process should be made to ensure the balance and effectiveness of the integrated approach.

**Response 7:**

**The original question:**

*While the method's effectiveness will be demonstrated through the self-balancing two-wheeled vehicle project, how can you ensure that the success is not overly specific to this case and can be generalized to other product types?*

**The authors’ answer:**

Firstly, the Kano, Axiomatic Design, and TRIZ methods can be individually applied to their respective domains. Kano is adept at analyzing user needs. Axiomatic Design is characterized by systematically analyzing design contradictions and evaluating design solutions. TRIZ stimulates innovative thinking and provides innovative problem-solving methods. The effectiveness of each method has been validated in various types of product design processes [16-20]. Secondly, the proposed method aligns with the general flow of product innovation design and does not include specific additional stages or steps. Lastly, different product types possess distinct characteristics. Therefore, when employing these methods, it may be necessary to make adjustments based on the specific circumstances.

In conclusion, the proposed method in this study can theoretically be applied to the design innovation of other types of products. However, in practical application, some level of adjustment to the method may be required based on the real-world situation.

**Response 8:**

**The original question:**

*TRIZ's toolbox is dynamic and evolves. How do you ensure that your method stays up-to-date with the latest TRIZ tools and techniques for problem-solving?*

**The authors’ answer:**

To ensure alignment with the latest tools and techniques for problem-solving in TRIZ, we actively engage in the TRIZ community and professional networks, including TRIZ workshops and training courses. Additionally, we actively seek out and track the latest TRIZ literature to stay updated on the newest tools and technologies. Furthermore, we collaborate and exchange knowledge with other TRIZ experts to share experiences and learn about the latest methods. By continuously learning and updating our knowledge, we can stay synchronized with the latest developments in TRIZ.

**Response 9:**

**The original question:**

*As technological advancements continue, how do you plan to evolve the integrated method to incorporate emerging theories or frameworks that might enhance product innovation?*

**The authors’ answer:**

Firstly, we regularly study the latest innovation theories and frameworks to understand their potential for problem-solving and promoting innovation. For example, we may focus on emerging approaches and tools in design thinking, agile development, and artificial intelligence.

Secondly, we compare and analyze the emerging theories with the integrated methods of Kano, Axiomatic Design, and TRIZ to determine their complementarity and potential for solving specific problems. If an emerging approach or framework can complement and expand the existing integrated methods, we will try to integrate it into the method.

Lastly, we continuously test and validate the updated integrated method through practice and case studies to assess its effectiveness and applicability. By continually incorporating new theories and frameworks into the integrated approach and validating them, we can make the method remain up-to-date and continuously evolve to adapt to the ever-changing innovation environment.

**Reference:**

[1] Du Laihong, Chen Hua, Fang Yadong, Liang Xiaowei, Zhang Yujie, Qiao Yidan, and Guo Zhen. 2022. "Research on the Method of Acquiring Customer Individual Demand Based on the Quantitative Kano Model." *Computational Intelligence and Neuroscience* 2022:5052711. doi: <https://doi.org/10.1155/2022/5052711>.

[2] Suh Nam Pyo. 2001. *Axiomatic Design: Advances and Applications*. New York, USA: Oxford University Press.

[3] Shirwaiker Rohan A., and Okudan Gül E. 2008. "Triz and axiomatic design: a review of case-studies and a proposed synergistic use." *Journal of Intelligent Manufacturing* 19 (1):33-47. doi: <https://doi.org/10.1007/s10845-007-0044-6>.

[4] Deng Xinyang, and Jiang Wen. 2018. "An Evidential Axiomatic Design Approach for Decision Making Using the Evaluation of Belief Structure Satisfaction to Uncertain Target Values." *International Journal of Intelligent Systems* 33 (1):15-32. doi: <https://doi.org/10.1002/int.21929>.

[5] Russo D., Rizzi C., and Montelisciani G. 2014. "Inventive guidelines for a TRIZ-based eco-design matrix." *Journal of Cleaner Production* 76:95-105. doi: <https://doi.org/10.1016/j.jclepro.2014.04.057>.

[6] Wu Xinping, Geng Xinzhou, Chen Zhiyi, Dong Aidi, and Li Jinchao. 2022. "Dynamic Design Quality Evaluation of Power Enterprise Digital System Based on Fuzzy Information Axiom." *Mathematical Problems in Engineering* 2022:2617640. doi: <https://doi.org/10.1155/2022/2617640>.

[7] Zou Fanxing, Zhu Xinyu, Qian Yuning, and Chang Danni. 2022. "A Sustainable Product Design Approach Based on Data Mining of Dynamic User Demands: A Case Study on HUAWEI Mate 40 Series." *IFAC-PapersOnLine* 55 (10):1056-1061. doi: <https://doi.org/10.1016/j.ifacol.2022.09.529>.

[8] Jin Hui Ying, Guo Haixia, Cao Guozhong, Wei Saina %J Applied Mechanics, and Materials. 2013. "Function Innovation Design Model and its Application in Heating Equipment." *Applied Mechanics and Materials* 401-403:371 - 374. doi: <https://doi.org/10.4028/www.scientific.net/AMM.401-403.371>.

[9] Gero John S., and Kannengiesser Udo. 2004. "The situated function–behaviour–structure framework." *Design Studies* 25 (4):373-391. doi: <https://doi.org/10.1016/j.destud.2003.10.010>.

[10] Zhiguo Zhang. 2009. "Research on product design based on the Axiomatic design." 2009 IEEE 10th International Conference on Computer-Aided Industrial Design & Conceptual Design, 26-29 Nov. 2009.

[11] Dou Runliang, Zhang Yubo, and Nan Guofang. 2017. "Iterative product design through group opinion evolution." *International Journal of Production Research* 55 (13):3886-3905. doi: <https://doi.org/10.1080/00207543.2017.1316020>.

[12] Liu W., and Hu C. 2018. "The Study of Interest Demands and Realization of Online Media User in Big Data Era." 2018 International Joint Conference on Information, Media and Engineering (ICIME), 12-14 Dec. 2018.

[13] Zhai Dongsheng, Li Mengyang, and Cai Wenhao. 2020. "TRIZ Technical Contradiction Extraction Method Based on Patent Semantic Space Mapping." 2020 The 11th International Conference on E-business, Management and Economics, Beijing, China.

[14] Liu T. L., Lu M. C., and Chen J. W. 2017. "A strategic planning of TRIZ applications for cloud-based collaborative product development." 2017 4th International Conference on Industrial Engineering and Applications (ICIEA), 21-23 April 2017.

[15] Zhang Peng, Ma Qianhao, Nie Zifeng, and Li Xindi. 2022. A Conflict Solving Process Based on Mapping between Physical Parameters and Engineering Parameters. *Machines* 10 (5): 323. doi:<https://doi.org/10.3390/machines10050323>.

[16] Deng Lujie, Romainoor Nurul H., and Zhang Bolun. 2023. Evaluation of the Usage Requirements of Hospital Signage Systems Based on the Kano Model. *Sustainability* 15 (6). doi:<https://doi.org/10.3390/su15064972>.

[17] Lin Qinqin, Wang Baihui, Zhang Xin, Wang Yawei, Zhao Hua, and Yang Jiaqi. 2023. A Study on the Demands of Physical and Medical Integration Services for the Elderly in the Dabei Quhou Community in Qingdao. *Sustainability* 15 (13). doi:<https://doi.org/10.3390/su151310064>.

[18] Yang Chen, and Zhang Xiufen. 2023. "Multi-domain Mapping design Method of Automatic Disassembly Equipment for Small Electromechanical Products." *Proceedings of the Institution of Mechanical Engineers, Part C: Journal of Mechanical Engineering Science*:09544062231181835. doi: <https://doi.org/10.1177/09544062231181835>.

[19] Liu Qinghua, Chen Jiadui, Yang Kai, Liu Dan, He Ling, Qin Qing, and Wang Yuqing. 2023. "An integrating spherical fuzzy AHP and axiomatic design approach and its application in human–machine interface design evaluation." *Engineering Applications of Artificial Intelligence* 125:106746. doi: <https://doi.org/10.1016/j.engappai.2023.106746>.

[20] Yao Kai-Chao, Huang Wei-Tzer, Xu Jing-Ran, Huang Shu-Hua, Tsai Chin-Tang, Ho Wei-Sho, and Liao Chun-Chung. 2023. Application of the TRIZ Innovation System Method to Bicycle Handlebars. *Machines* 11 (5). doi:<https://doi.org/10.3390/machines11050507>.

**Part Ⅱ**

**Part Ⅱ** covers Page **10** to Page **41**, which includes responses to Reviewer 2 and relevant references.

Throughout the following response, we will adhere to the following format:

Firstly, each response will consist of three parts: the original comments from the reviewer, the summary of the comments from the authors, and the response from the authors. To better differentiate the text content, the original comments will be in italics, while the answers will not.

Additionally, as the reply encompasses modifications to the manuscript, the authors’ response will include a summary of the modifications made and the specific changes and additions incorporated into the manuscript. Notably, to emphasize the changes made to the original version, the original one will be presented in black font, while modifications and additions will be presented in blue font.

Finally, the references used during the answering process will be presented upon completing all question responses.

**Response 1:**

**The original comments:**

*Would you explicitly specify the novelty of your paper? What progress against the most recent state-of-the-art similar studies was made?*

*The organization of the Introduction section is very unsatisfactory, and it is very messy and hard to read. Thus, this section needs rewriting in order to make it crisp and the main points of the research methodology should be mentioned clearly. This will help the readers to appreciate the novelty of the research.*

*You have to sharply delineate your research question and gap. Please, be more aware of deriving the question out of the relevant literature streams.*

*More state-of-the-art methods (models + algorithms) must be reviewed and compared to the suggested ones in this work. I suggest the authors find and read the studies performed by scholars such as Goli et al. and their groups in this regard.*

**The summary of the comments:**

We need to elucidate the critical novel contributions of our study and illustrate the progress made through a comprehensive review of the relevant literature. Furthermore, attention should be paid to the research conducted by the Goli et al. team when reviewing the literature.

**The authors’ answer：**

The summary of the modifications made：

Firstly, adjustments have been made to the content of the methodology and case study sections in the original manuscript. The modifications in the methodology and case study sections are as follows: the content discussing the evaluation of the proposed solution, initially found in the discussion section, has been moved to the methodology section. This change also led to corresponding adjustments in the case study section.

Secondly, we have redefined the novelty of the research, resulting in a rewriting of the introduction section, as well as the abstract and conclusion of the article. The new introduction explicitly states the key innovative aspects of the paper and highlights the points of differentiation from existing studies. The revised abstract and conclusion will be presented in Response 2, so they will not be emphasized here.

Additionally, the revised manuscript has reorganized certain parts of the literature review section. Furthermore, a comprehensive review of the latest literature has been included to highlight the research gap. These additions will be reflected in the new literature review section.

Last but not least, we have optimized the manuscript by improving the clarity of expression while modifying the content.

To facilitate the reviewer and prevent him from continuously switching between the "Response to Reviewers" and "Revised Manuscript with Track Changes" files, the modifications will be presented comprehensively and precisely, following the sequence of introduction, literature review, methodology, and case study sections.

The specific changes and additions：

**Introduction**

~~To enhance the competitiveness of companies in modern society, it is necessary to improve the efficiency and innovation of product design [1]. Faced with diverse market demands and complex product functionalities and structures, designers are required not only to accurately identify user needs at the early stage of design projects but also to be skilled in analyzing design problems and possess problem-solving capabilities. AD is a method that studies design problems from a systemic perspective. It establishes a mapping relationship between user needs, functional requirements, design elements, and manufacturing processes, which helps designers transform functional requirements into design elements and identify contradictions between functional requirements and design elements [2]. However, this theoretical approach has two obvious limitations. Firstly, AD theory does not provide an accurate method for identifying user needs, resulting in a lack of a reliable basis for the mapping between user needs and functional requirements. Secondly, AD does not offer a problem-solving method, requiring designers to find reasonable solutions through trial and error. These limitations restrict the application of AD theory. Kano is an effective method for identifying user needs and prioritizing them [3]. TRIZ is a tool that helps generate creative ideas and solve design problems [4]. While some scholars have recognized the limitations of the problem-solving capabilities of AD theory and have combined AD with TRIZ, few have paid attention to the subjectivity of AD when analyzing user requirements. The methods and processes proposed by these scholars are consistently incomplete for any design project. This paper proposes a method that integrates Kano and TRIZ with AD, aiming to address the limitations of AD theory. Furthermore, it ensures the completeness of the product design process, making it more convenient for designers to accomplish a comprehensive design project. The effectiveness of the new process is demonstrated through a design practice.~~

~~The main contributions of this study are as follows. Firstly, the paper integrates Kano and TRIZ within the framework of AD theory, addressing the limitations of axiomatic design and enriching and expanding the AD theory. Secondly, the innovative product design method proposed in the manuscript ensures the completeness of the design process, providing convenience for designers to accomplish a comprehensive design project. Finally, the research case presented in the manuscript has successfully applied for a new patent and provides new directions for the design improvement of the self-balancing two-wheeled vehicle.~~

In the context of fast-paced product iteration, the design innovation capability of companies needs to be enhanced [1]. So, the design innovation methods and processes must be effective and as simple as possible. To propose practical methods, it is necessary to outline the basic design innovation process. Improving user satisfaction is the ultimate goal of continuous product innovation [2]. Therefore, better services and products must start by analyzing user needs [3]. User needs determine the product's functional attributes, and implementing these functions relies on technical principles and product structure [4]. Analyzing the mapping relationship between functions and structure and resolving conflicts are the main tasks of product functional design [5]. After completing the design solution, evaluating whether the product has been improved and optimized is necessary, which will help designers make design decisions [6]. In summary, the complete process of product innovation design includes five main steps: user needs research (S1), requirement-function transformation (S2), analysis of design issues (S3), resolution of design issues (S4), and design solution evaluation (S5).

Previous studies have proposed methods that only cover a single or a few steps in the product design process. These methods cannot guide the entire process of product innovation design. Further literature research has revealed that some scholars have paid attention to the need for strategies that can guide the whole process. However, these methods required the integration of more than three theories, which brought forth other issues. Readers needed a broad theoretical foundation to comprehend research articles and the design process. Additionally, the abundance of methods increased the complexity of the design practice, making it challenging for readers to learn and difficult to apply these methods widely.

The paper proposed an integrated method that covered the entire design process, utilizing only three theoretical approaches. This new method ensures guidance throughout the design workflow while reducing the complexity of both theory and practice. The proposed approach involves the use of Kano analysis for understanding user requirements (S1), utilizing the AD theory for requirement function transformation, design problem analysis, and design solution evaluation (S2, S3, S5), and incorporating TRIZ for design problem-solving (S4). The paper demonstrated the new process's effectiveness through a design practice.

**Literature review**

~~This section is a literature review that introduces the basic theories and research applications of AD, Kano, and TRIZ methods, and explains their advantages and disadvantages.~~ This literature review introduces the basic theories and research applications of Kano, AD, and TRIZ methods, their advantages and disadvantages, and the research gap.

**~~Axiomatic design~~ Kano**

~~Axiomatic Design (AD) was initially proposed by Professor Suh in 1976 [5-6]. This theoretical approach aims to establish design specifications in a quantitative manner, assisting designers in enhancing the logic and systematics of problem analysis [7]. In the axiomatic theory, all objects involved in the design process are classified into four categories: users, functions, structures, and processes. The sets consisting of multiple objects of the same category are defined as domains, namely Customer Attributes domain (CAs), Functional Requirements domain (FRs), Design Parameter domain (DPs), and Process Variables domain (PVs) [8]. There exists a mapping relationship between these domains in a zigzag pattern, as shown in Fig 1. After clarifying the relationships between the elements, AD theory analyzes design problems based on two axioms: the independence axiom and the information axiom [9-10]. AD utilizes design matrices and equations to express the relationship between the functional domain and the design parameter domain. It can be classified into non-coupled design, decoupled design, and coupled design [11]. The occurrence of the coupled design indicates problems or contradictions between functions and structures, requiring designers to find ways to decouple them [12]. The mapping relationships constructed in axiomatic design demonstrate the problems in design projects, enabling designers to analyze design problems from a systemic perspective [13-14]. However, previous research has indicated two notable limitations of axiomatic design. Firstly, although the concept of the customer domain is introduced, no precise method is provided for accurately identifying user needs and classifying their priorities [15]. Secondly, the ability to provide suitable and appropriate design suggestions is insufficient when presenting concrete solutions [16]. These are the main reasons why this paper proposes the integration of Kano, TRIZ, and AD theory.~~

Kano, proposed by Professor Noriaki Kano, is a two-dimensional cognitive model that studies the nonlinear relationship between product quality performance and user satisfaction [7]. The Kano model is widely applied in product development to help designers clarify user needs and establish product objectives [8]. The theoretical model classifies user needs into five categories based on the relationship between the completeness of functional requirements and user satisfaction: Must-Requirement (M), One-Dimensional Requirement (O), Attractive Requirement (A), Indifferent Requirement (I), and Reverse Requirements (R) [9-12]. These classification criteria assist in identifying explicit and implicit user needs, which contribute to designing new products or improving existing ones [13]. AD theory lacks specific methods for discovering and analyzing user needs. Kano can compensate for AD's shortcomings and enhance the accuracy of mapping user needs to functional requirements.

**~~Kano~~ Axiomatic design**

~~Kano, proposed by Professor Noriaki Kano, is a two-dimensional cognitive model used to study the nonlinear relationship between product quality performance and user satisfaction [17]. The Kano model is widely applied in product development to help designers clarify user needs and establish product objectives [18]. In this theoretical model, user needs are classified into five categories based on the relationship between the completeness of functional requirements and user satisfaction: Must-Requirement (M), One-Dimensional Requirement (O), Attractive Requirement (A), Indifferent Requirement (I), and Reverse Requirements (R) [19-22]. These classification criteria assist in identifying both explicit and implicit user needs and prioritizing them. This can be utilized in designing new products or improving existing ones [23]. AD theory lacks specific methods for discovering and analyzing user needs. Kano can compensate for the shortcomings of AD and improve the accuracy of mapping user needs to functional requirements.~~

Professor Suh proposed Axiomatic Design (AD) in 1976 [14,15]. This theoretical approach aims to establish design specifications quantitatively, assisting designers in enhancing the logic and systematics of problem analysis [16]. The theory proposes the concepts of customer domain (CAs), functional domain (FRs), structural domain (DPs), and process domain (PVs) to standardize the design process [17]. A mapping relationship exists between these domains in a zigzag pattern, as shown in Fig 1. AD utilizes design matrices to express the relationships between the functional and structural domains and employs the independence axioms to analyze design problems [18-19]. When there is a coupled design, it indicates a contradiction between function and structure, and designers need to find ways to decouple them [20-21]. After obtaining design solutions, the information axioms in AD help designers make decisions by quantitatively comparing the information content in design alternatives [22]. Therefore, AD can assist in analyzing problems and evaluating solutions. However, previous research has indicated two notable limitations of AD. Firstly, although AD introduces the concept of the customer domain, it fails to provide an accurate method for identifying user needs and classifying their priorities [23]. Secondly, the ability to provide appropriate design suggestions is insufficient when presenting concrete solutions [24]. These are the main reasons the paper proposes the integration of Kano, TRIZ, and AD.

**Fig 1. The Z-shaped mapping relationship between the domains**

**Theory of inventive problem solving**

Theory of Inventive Problem Solving(TRIZ) has been widely and rapidly adopted in academic and industrial fields as an efficient method for solving conflicting problems [25]. In 1946, Altshuller proposed TRIZ~~, which is~~ based on the analysis of interdisciplinary invention patents [26]. ~~And~~ Since then, llevbary et al. have developed the complete theoretical system of TRIZ by proposing the contradiction matrix, 40 principles of the invention, and other related theoretical models, tools, and research methods [27]. TRIZ summarizes innovation methods from all fields and proposes a universal pattern that can be applied to engineering, tourism, catering, sharing economy, e-commerce, and other areas [28]. By addressing problems at the system level, TRIZ can effectively balance different interests in product design [29], and many designers and product design scholars have achieved effective design results using TRIZ [30,31].

The researcher classified all the tools and methods in the TRIZ theory system into three levels: philosophy, principles, and tools [32,33], as shown in Fig 2. In actual use, researchers can flexibly choose the required methods based on the specific situation. Using contradictions to define problems and solve them with a contradiction matrix is one of the most commonly used methods in TRIZ theory [34]. ~~Two types of contradictions are defined in TRIZ theory: technical contradictions and physical contradictions.~~ TRIZ theory describes two types of contradictions: technical and physical contradictions. When a technical contradiction arises, changing one attribute in the system will lead to the deterioration of ~~other attributes~~ others. When a physical contradiction arises, the same parameter in the system cannot exist under different requirements [35]. ~~By~~ Inventive principles will be found by analyzing the contradictions ~~that arise~~ and comparing them to the corresponding engineering parameters and contradiction matrix~~, inventiveness principles that can solve the problem will be found~~ [36]. Many published studies have demonstrated the effectiveness of this method [37-39]. For designers, the methods in TRIZ theory can provide new design inspiration and help solve contradictions that arise during the design process [40,41]. ~~After analyzing design problems using AD, TRIZ can be utilized to help solve these problems and compensate for the lack of solution methods in AD theory.~~ After analyzing design problems using AD, designers can utilize TRIZ to help solve these problems, which will compensate for the lack of solution methods in AD.

**Fig 2. Summary and classification of TRIZ theory**

**Research gap**

This study presents the literature through tables to provide a detailed overview of the existing research status. Due to the extensive literature volume, the tables only include research papers that meet the following two criteria: i. The papers were published within the past five years; ii. The papers proposed methodological processes that covered at least two steps in the design process. Table 1 displays the correspondence between the full names and abbreviations of theoretical methods, while Table 2 presents the relevant references.

Numerous scholars have proposed improved methods for individual or multiple steps in product innovation design. Reference [42] examined the current needs of users using Kano (S1). References [43,44] utilized mathematical models and algorithms to predict future product demands (S1). Reference [45] established the connection between user requirements and product functions by QFD (S2). Additionally, it introduced a robot concept design for architectural layouts through TRIZ (S4). References [46-48] analyzed contradictions between functionalities and structures with AD (S3) and subsequently proposed design solutions by applying TRIZ (S4). Reference [50] constructed an evaluation system through literature research and the entropy evaluation method and evaluated mobile applications in government services by grey relational analysis (S5). Reference [52] employed qualitative methods to determine user requirements for bicycle handlebars (S1). Then, it proposed solution strategies using TRIZ (S4) and evaluated user satisfaction with improved handlebars by utilizing Kano and IPA (S5). These methodological processes presented in the studies can effectively enhance the efficiency or accuracy of specific steps in the design process, but they did not provide guidance for the entire process of product innovation design.

Further literature research reveals that some scholars have noticed the research gap in existing methods that failed to cover the entire innovation design process. To address this issue, they have integrated multiple theoretical methods. In reference [59], the KJ method and Kano survey were employed to analyze user requirements for Smart Neck Helmets (S1). And QFD was used to prioritize the importance of functional requirements and engineering parameters, providing a basis for analyzing design conflicts (S2, S3). Subsequently, TRIZ was applied to propose a new design solution (S4). Finally, the scheme was evaluated by FEA (S5). In reference [60], FMEA was utilized to capture user requirements for pressure relief valves. Then, QFD was employed to convert user requirements into functional and technical requirements, and TRIZ was used to develop a new valve structure solution. The solution was evaluated using LCA and Fuzzy TOPSIS. Although the studies guided the entire process of product innovation design, they necessitated the utilization of five different research theories and methods, which had potential drawbacks. First, it required readers to have a broad theoretical foundation to understand the design process and comprehend the research articles. Moreover, the multitude of methods increased the complexity of the design practice, which limited the practical application of these methods.

The paper proposed a new product innovation design process. This new process has two advantages. Firstly, this method can guide the entire process of product innovation design. Additionally, the new process incorporates only three theoretical methods, greatly reducing the learning burden for readers, while also lowering the difficulty and complexity of design practice.

**Table 1. The full names and abbreviations of the theoretical methods**

| **Full Name** | **Abbreviation** | **Full Name** | **Abbreviation** |
| --- | --- | --- | --- |
| Axiomatic Design | AD | Important-Performance Analysis | IPA |
| Analytic Hierarchy Process | AHP | Kano | Kano |
| Algorithm for  Inventive-Problem Solving | ARIZ | KJ Method | KJ |
| Design Structure Matrix | DSM | Life Cycle Evaluation | LCE |
| Decision Making Trial and Evaluation Laboratory | DEMATEL | Principal Component Analysis | PCA |
| Evaluation Grid Method | EGM | PUGH Concept Selection Matrix | PCM |
| Function Analysis System Technique | FAST | Quantification Theory Type I | QTT1 |
| Failure Mode and Effects Analysis | FMEA | Quality Function Deployment | QFD |
| Finite Element Analysis | FEA | Quality Function Deployment Combined with Environmental Aspects | QFDE |
| Grey Relational Analysis | GRA | Semantic Difference | SD |
| House of Quality | HOQ | Technique for Order Preference by Similarity to an Ideal Solution | TOPSIS |
| Interpretive Structural Modeling | ISM | Theory of Inventive Problem Solving | TRIZ |

**Table 2. The references on research related to product innovation design**

| **Number of Methods** | **Reference** | **Tool used** | **S1-S5** | | | | | **Brief Description** |
| --- | --- | --- | --- | --- | --- | --- | --- | --- |
|  |  |  | **S1** | **S2** | **S3** | **S4** | **S5** |  |
| Two | 45 | QFD/TRIZ |  | √ | √ | √ |  | A conceptual robot design for an automated layout of building structures was proposed using QFD and TRIZ. |
|  | 46 | AD/TRIZ |  |  | √ | √ |  | The paper designed a cutter-changing robot using modular design, incorporating AD and TRIZ. |
|  | 47 | AD/TRIZ |  |  | √ | √ |  | The paper helped designers utilize AD and TRIZ under incomplete information environments. |
|  | 48 | AD/TRIZ |  |  | √ | √ |  | In modular design, a new design approach for module interfaces was proposed by AD and TRIZ. |
|  | 49 | Kano/QFD | √ | √ | √ |  |  | The paper utilized Kano and QFD to identify user requirements and design elements, proposing an optimized design for wooden office desks. |
| Three | 51 | Kano/QFDE/TRIZ | √ | √ |  | √ |  | The paper utilized Kano and QFD to identify requirements and proposed product structures by TRIZ. |
|  | 52 | TRIZ/Kano/IPA | √ |  |  | √ | √ | The paper qualitatively determined user requirements and employed TRIZ to conduct an improvement design of bicycle handlebars. Kano and IPA evaluated the new solution. |
|  | 53 | AHP/QFD/PCM | √ | √ | √ |  | √ | The paper utilized AHP and QFD to analyze the needs of surgical healthcare professionals and the design characteristics of medical devices. PCM evaluated the design proposals. |
|  | 54 | Kano/TRIZ/QFD | √ | √ | √ | √ |  | The paper identified user requirements and functional technical requirements with Kano and QFD. TRIZ was employed to propose design solutions for a four-wheel vehicle. |
|  | 55 | Kano/QFD/PCM | √ | √ | √ |  | √ | The paper employed Kano and QFD to identify user requirements and design factors and utilized PCM to evaluate design solutions. |
|  | 56 | QFD/AHP/ARIZ | √ | √ | √ | √ |  | The paper applied AHP and QFD to analyze the user requirements and technological features of disinfection and epidemic prevention robots. ARIZ proposed the design scheme. |
|  | 57 | EGM/GRA/QFD | √ | √ | √ |  |  | The paper acquired user requirements for wickerwork lamps by EGM and GRA. QFD transformed the requirements into technical characteristics, leading to new design solutions. |
| Three or more Varieties | 58 | Kano/HOQ/QFD/TRIZ/ISM/DSM | √ | √ | √ | √ |  | The innovative design of temporary refugee housing was achieved by employing the combined methods in this study. |
|  | 59 | KJ/Kano/QFD/TRIZ/FEA | √ | √ | √ | √ | √ | The paper identified user and functional technical requirements by KJ, Kano, and QFD. TRIZ proposed the solutions, evaluated by FEA. |
|  | 60 | FMEA/QFD/TRIZ/LCA/TOPSIS | √ | √ | √ | √ | √ | The paper employed FMEA and QFD to analyze user and technical requirements. Design solutions were proposed by TRIZ and evaluated through LCA and TOPSIS. |
|  | 61 | SD/PCA/TRIZ/AHP | √ |  |  | √ | √ | The paper employed SD and PCA to analyze user requirements, proposed design solutions using TRIZ and evaluated the solutions by AHP. |
|  | 62 | Kano/AHP/DEMATEL/QFD | √ | √ | √ |  |  | The paper employed Kano and AHP to investigate user requirements. DEMATE and QFD established the relationship between user requirements and design factors. |
|  | 63 | Kano/AHP/QFD/PCM | √ | √ | √ |  | √ | The paper identified user requirements with Kano and AHP, transformed the requirements into functional requirements using QFD, and evaluated the proposed solution by PCM. |
|  | 64 | EGM/QTT1/TRIZ/AHP | √ |  |  | √ | √ | The paper employed EGM and QTT1 to analyze user requirements, proposed design solutions using TRIZ and evaluated the solutions by AHP. |
|  | 65 | KJ/Kano/FAST/QFD | √ | √ | √ |  |  | The paper identified user requirements with KJ and Kano, decomposed the product functionalities using FAST, and applied QFD to establish the correlation between user needs and product features. |

**Method**

The theoretical approach proposed in this paper consists of ~~five~~ six main parts: clarifying user requirements, establishing a product function model, analyzing the conflicts between functional requirements and design parameters, resolving the conflicts based on TRIZ, ~~and~~ validating and refining the design solution, and evaluating the scheme. The process is illustrated in Fig 3.

**Fig 3. The innovation design process based on Kano, AD, and TRIZ**

**Clarify user requirements**

~~The Kano model is applied in a sequential manner, following the steps of designing the questionnaire, collecting responses, and processing data to identify user needs and classify them into different types.~~ The Kano model is applied to identify user needs and classify them into different types, following the steps of designing the questionnaire, collecting responses, and processing data. This step provides a basis for ~~the~~ mapping ~~of~~ user needs to functional requirements and enhances accuracy.

**Establish the function model**

After clarifying user needs through the Kano model, in order to improve the accuracy of mapping functional requirements to design parameters, it is necessary to have a systematic understanding of the structural principles of the product. The paper will establish a functional model to decompose the functional structure of the product [66]. The functional model describes the relationships and interactions between components or parts of the product in a graphical manner [67], as shown in Fig 4.

**Fig 4. Establish the function model**

**Analyze contradictions between product functions and design parameters**

This process consists of three main steps.

Firstly, the functional requirements are decomposed based on the user needs identified by Kano.

Secondly, the mapping relationship between functional requirements and design parameters is established based on the functional model, as shown in Table ~~1~~3. The design equation can be represented as Eq. (1), where X indicates the influence of the structure on the function, and O indicates no influence of the structure on the function.

**Table ~~1~~3. Build the mapping relationship between Functional Requirements and Design Parameters**

| **Functional Requirements (FRs)** | **Design Parameters (DPs)** |
| --- | --- |
| FR_1_ | DP_1_ |
| FR_2_ | DP_2_ |

$$\left\{ \begin{aligned} {FR}_{1} \\ {FR}_{2} \end{aligned} \right\}=\left[ \begin{aligned} X O \\ O X \end{aligned} \right]\cdot\left\{ \begin{aligned} {DP}_{1} \\ {DP}_{2} \end{aligned} \right\}$$

(1)

Thirdly, identify contradictions between product functional requirements and design parameters. According to the independence axiom in AD theory, the coupling of the design matrix is determined. ~~Taking~~ Take Eq. (2) as an example. The form of the matrix indicates that it is a coupling matrix. ~~And there exist three groups of conflicts between functional requirement and design parameters, namely FR1 and DP3, FR3 and DP1, and FR3 and DP2.~~ Three groups of conflicts exist between functional requirements and design parameters, namely FR_1_ and DP_3_, FR_3_ and DP_1_, and FR_3_ and DP_2_. To decouple the matrix, these three conflicts are required to be solved.

$$\left\{ \begin{aligned} {FR}_{1} \\ {FR}_{2} \\ {FR}_{3} \end{aligned} \right\}=\left[ \begin{aligned} X O X \\ O X O \\ X X X \end{aligned} \right]\cdot\left\{ \begin{aligned} {DP}_{1} \\ {DP}_{2} \\ {DP}_{3} \end{aligned} \right\}$$

(2)

**Resolve conflicts based on TRIZ**

The process mainly consists of three steps.

Firstly, complete the conversion of general parameters. In this step, the product components involved in the contradiction between function and structure need to be transformed into engineering parameters according to the definition in TRIZ theory, and the improvement factors and deterioration factors should be identified.

Secondly, select appropriate inventive principles and resolve contradictions. Based on the results of factor transformation in Step 1, refer to the Achshuler conflict matrix to find the inventive principle corresponding to the intersection of the improved ~~parameter~~ and deteriorated parameters. Compare the obtained inventive principles with the design objectives and select the appropriate ones to resolve the design conflicts.

**Validate and refine the design scheme**

Verify the design scheme using the independence axiom in AD theory to determine its rationality. If the design scheme is deemed reasonable, further improvements can be made to enhance its effectiveness.

**Evaluate the design scheme**

After confirming that the design scheme complies with the independent axioms of AD and refining the design scheme, evaluate the design scheme by the information axioms in AD, comparing the amount of information in the scheme with the original product. Firstly, convert the user requirements obtained through Kano analysis into evaluation system indicators. Secondly, divide the evaluation levels of each indicator and determine the design scope based on user requirements. Thirdly, evaluate the scheme through expert evaluation. Finally, take the triangular fuzzy function as the membership function to convert linguistic terms into corresponding fuzzy numbers [68], draw the function graph, and calculate the amount of information according to Eq. (3).

$$I={log}_{2}\left( \frac{fuzzy design scope}{fuzzy public scope} \right)$$

(3)

**Case study**

The self-balancing two-wheeled vehicle, as a means of transportation, has gained popularity among people in modern society. ~~For college students, it has become a common sight to see them using self-balancing two-wheeled vehicles to travel around the campus.~~ It has become common for college students to see them taking self-balancing two-wheeled vehicles to travel around the campus. This paper takes the design of a self-balancing two-wheeled vehicle as a practical case study to demonstrate the feasibility and effectiveness of the new process.

**Identify user requirements for the self-balancing two-wheeled vehicle**

Through expert evaluation, the user requirements for the self-balancing two-wheeled vehicle are determined ~~using~~ by Kano analysis ~~in terms of~~ regarding commuting functionality, safety, comfort, portability, maintainability, and aesthetics. Based on the final calculation results, Must-Requirements include "ease of standing on the vehicle", "stable structures", and "responsive braking system". One-Dimensional Requirements include "simplicity of structure for easy maintenance" and "aesthetically pleasing appearance". Attractive Requirements include "good shock absorption", "lightweight", "handle for carrying", and "reduced size when idle". Indifferent Requirement is "riding speed". All the calculation results are presented in Table 4.

**Table 4. Analysis of user requirements in Kano**

| **The first-level user requirements** | **The second-level user requirements** | **Kano attributes** | **Better coefficient** | **Worse coefficient** |
| --- | --- | --- | --- | --- |
| **Commuting functionality** | Ease of standing on the vehicle | M | 11.90% | -88.10% |
|  | Riding speed | I | 35.71% | -26.19% |
| **Safety** | Stable structures | M | 11.9% | -95.24% |
|  | Responsive braking system | M | 16.67% | -80.95% |
| **Comfort** | Good shock absorption | A | 83.33% | -40.48% |
| **Portability** | Lightweight | A | 80.95% | -19.05% |
|  | Handle for carrying | A | 61.9% | -11.9% |
|  | Reduced size when idle | A | 83.33% | -7.14% |
| **Maintainability** | Simple structures for easy maintenance | O | 45.24% | -57.14% |
| **Aesthetics** | Aesthetically pleasing appearance | O | 64.29% | -59.52% |

**Establish the functional model of the self-balancing two-wheeled vehicle**

Through function analysis, the structural principles of the two-wheeled balancing vehicle, as well as the relationships and interactions among its components, can be determined, as shown in Fig 5. This lays the foundation for establishing the mapping relationship between FRs and DPs.

**Fig 5. Establish the function model of a self-balancing two-wheeled vehicle**

**Analyze the conflicts between the functional requirements and design parameters**

Firstly, decompose the functional requirements based on the user requirements identified by Kano, as shown in Fig 6.

Secondly, establish the mapping relationship between functional requirements and design parameters based on the functional model. Refer to Table 5 for details.

**Fig 6. The decomposition of the functional requirements**

**Table 5. The mapping relationship between Functional Requirements and Design Parameters**

| **Functional Requirements (FRs)** | **Design Parameters (DPs)** |
| --- | --- |
| Ease of standing on the vehicle(FR_11_) | Footboard area(DP_11_) |
| Riding speed(FR_12_) | Transmission structure and wheels(DP_12_) |
| Stable structures(FR_21_) | Car frame (DP_21_) |
| Responsive braking system(FR_22_) | Gyroscope and controller(DP_22_) |
| Good shock absorption (FR_31_) | Suspension structure(DP_31_) |
| Lightweight(FR_41_) | Material (DP_41_) |
| Handle for carrying(FR_42_) | Lifting structure(DP_42_) |
| Reduced size when idle (FR_43_) | Folding structures(DP_43_) |
| Simple structures for easy maintenance (FR_51_) | Car frame(FR_51_) |
| Aesthetically pleasing appearance (FR_61_) | Appearance (FR_61_) |
| Ease of standing on the vehicle(FR_11_) | Footboard area(DP_11_) |

Thirdly, analyze the conflicts between functional requirements and design parameters based on the independence axiom. According to the mapping results in Table 5, the equations between portable self-balancing scooter functional requirements and product structure are listed, resulting in the design equation as shown in Eq. (4). From the design matrix, it can be observed that there are 5 groups of conflicts.

$$\left\{ \begin{aligned} {FR}_{11} \\ {FR}_{12} \\ {FR}_{21} \\ {FR}_{22} \\ {FR}_{31} \\ {FR}_{41} \\ {FR}_{42} \\ {FR}_{43} \\ {FR}_{51} \\ {FR}_{61} \end{aligned} \right\}=\left[ \begin{aligned} X O O O O O O O O O \\ O X O O O O O O O O \\ O O X O O X O X X O \\ O O O X O O O O O O \\ O O O O X O O O O O \\ O O O O O X O O O O \\ O O O O O O X O O X \\ X O O O O O O X O O \\ O O X O O O O X X O \\ O O O O O O O O O X \end{aligned} \right]\cdot\left\{ \begin{aligned} {DP}_{11} \\ {DP}_{12} \\ {DP}_{21} \\ {DP}_{22} \\ {DP}_{31} \\ {DP}_{41} \\ {DP}_{42} \\ {DP}_{43} \\ {DP}_{51} \\ {DP}_{61} \end{aligned} \right\}$$

(4)

There exists a conflict between "stable structures(FR_21_)" and "material(DP_41_)". Achieving structural stability requires high material strength, but high-strength materials may have a higher density, which would increase the weight of the self-balancing two-wheeled vehicle.

There exists a conflict between " stable structures(FR_21_)" and "folding structures(DP_43_)". While the inclusion of a folding structure may reduce the size when idle, it may also introduce movable components that compromise the structural stability.

There exists a conflict between "handle for carrying(FR_42_)" and "appearance(DP_61_)". Adding a handle would make it more convenient for users to carry the self-balancing two-wheeled vehicle, but it may affect the product's overall aesthetics.

There exists a conflict between " reduced size when idle (FR_43_)" and "footboard area (DP_11_)". Reducing the product size may result in a smaller footboard area, which could impact the riding experience for users.

There exists a conflict between "simple structures for easy maintenance(FR_51_)" and "folding structures(DP_43_)". The inclusion of a folding structure would increase the complexity of the vehicle's structure, making maintenance more challenging.

**Resolve design issues of the self-balancing two-wheeled vehicle based on TRIZ**

Firstly, convert the factors of the self-balancing two-wheeled vehicle into general engineering parameters. Extract the factors that need improvement and the factors that may worsen from the five groups of conflicts, and transform them into the general parameters, as shown in Table 6.

**Table 6. Factors transformation**

| **Serial number** | **Factors that need to be improved** | | **Factors that will worsen** | |
| --- | --- | --- | --- | --- |
|  | **Factors** | **General parameters** | **Factors** | **General parameters** |
| **1** | Stable structures | Stability of the structure(NO.13) | Weight | The weight of a stationary object(NO.2) |
| **2** | Reduced size when idle | Adaptability and versatility(NO.35) | Stable structures | Stability of the structure(NO.13) |
| **3** | Handle for carrying easily | Operability(NO.33) | Appearance | Shape(NO.12) |
| **4** | Reduced size when idle | The volume of a stationary object(NO.8) | Footboard area | The area of a moving object(NO.5) |
| **5** | Added folding structure | The number of materials or objects(NO.26) | Easy maintenance | Maintainability(NO.34) |

Secondly, select appropriate invention principles. Through the general parameters obtained from Table 6, the paper refers to the Achshuler conflict matrix to find the corresponding invention, as shown in Table 7. ~~Based on the conflict matrix, more than twenty principles of the invention were obtained.~~ More than twenty invention principles are obtained based on the conflict matrix. ~~After careful selection, principles of invention numbered 2, 5, 15, and 7 have been chosen as the guiding methods for designing the self-balancing two-wheeled vehicle.~~ After careful selection, the paper chooses principles of invention numbered 2, 5, 15, and 7 as the guiding methods for designing the self-balancing two-wheeled vehicle.

**Table 7. The invention principles found in the Achshuler conflict matrix**

| **Improved parameters** | **Deterioration parameter** | | | | |
| --- | --- | --- | --- | --- | --- |
|  | **The weight of a stationary object(NO.2)** | **Stability of the structure(NO.13)** | **Shape(NO.12)** | **The area of a moving object(NO.5)** | **Maintainability(NO.34)** |
| **Stability of the structure(NO.13)** | 26,29,1,40 | / | 22,1,18,4 | 2,11,13 | 2,35,10,16 |
| **Adaptability and versatility(NO.35)** | 9,15,29,16 | 35,30,14 | 15,37,1,8 | 35,30,29,7 | 1,16,7,4 |
| **Operability(NO.33)** | 6,13,1,25 | 32,35,30 | 15,34,29,28 | 1,17,13,16 | 12,26,1,32 |
| **The volume of a stationary object(NO.8)** | 35,10,19,14 | 34,28,35,40 | 7,2,35 | / | 1 |
| **The number of materials or objects(NO.26)** | 27,26,18,35 | 15,2,17,40 | 35,14 | 15,14,29 | 2,32,10,25 |

Thirdly, utilize the invention principles to resolve design issues.

Above all, according to the invention principle No.2, the principle of extraction means that removing the part or attribute that has a negative effect is extracted from the object. The power transmission shaft component in the power system is extracted, thereby reducing the complexity and difficulty of the folding wheel structure.

Next, the ~~principle of invention 5~~ invention principle No.5 is used for combination, which involves combining or merging the same objects or related operations in space or time. Combine the motor that generates power with the moving wheel, namely utilize hub motors to provide motive force for a self-balancing two-wheeled vehicle. The permanent magnet hub motor designed and researched by Ai Dong et al. can be a good choice, ~~which is~~ characterized by good stability, lightweight, and compact design [69].

Furthermore, the ~~principle of invention 15~~ invention principle No.15, the dynamic principle, is meant to divide an object and make each of its parts ~~can~~ alter its relative positions. ~~In order to~~ To achieve folding function, certain components of the self-balancing scooter are allowed to change their relative positions. During this process, it is necessary to set up two rotating mechanisms to achieve the folding of the footboards and the wheels. ~~To achieve rotational folding of the footboard, a hinge is used between the handle and the footboard. And the shape of the handle is designed to limit the rotation angle of the footboard, ensuring the stability of the product structure while riding.~~ A hinge is used between the handle and the footboard to achieve rotational folding of the footboard. The shape of the handle is designed to limit the footboard's rotation angle, ensuring the product structure's stability while riding. Moreover, it ~~is also ensured~~ ensures the footboards can be quickly and easily folded when users need to carry the vehicle. A connecting shaft is employed between the wheel with a hub motor and the footboard. To realize the folding of wheels, a fixed joint is utilized to connect the connecting shaft with the wheel, while a rotating joint is adopted to connect the connecting shaft with the footboard. The structure enables the wheel to rotate onto the two-dimensional plane where the footboard is located, further reducing the overall volume of the product after folding.

Eventually, the ~~principle of invention 7~~ invention principle No.7, the nesting principle, is employed to allow one object to pass through or be within the cavity of another object. To ensure the stability of the ~~structure of the vehicle~~ vehicle structure during riding and the simplicity of folding operation, a self-locking system needs to be designed. Two connecting shafts with different shape features are nested between the connecting shaft and the footboard to achieve dual functionality. During the operation of the vehicle, a square axis is used to connect the connecting shaft and the footboard to prevent relative displacement between the footboard and the wheel. When folding the self-balancing vehicle, the square axis is replaced with a round axis, so ~~that~~ the connecting shaft can act as a pivot. These two connecting shafts can be interchanged through buttons, card slots, and springs.

By comprehensively applying the inventive principles, a folding structure that meets the requirements is obtained. The schematic diagram of this structure is shown in Fig 7. As the two-wheeled self-balancing vehicle is ~~a~~ symmetrical ~~structure~~, only the right side is shown in the figure.

**Fig 7. The structural explosion diagram of the product in the design scheme**

**Validate and refine the design scheme**

Firstly, the rationality of the design proposal is validated using the independence axiom of the AD theory. The mapping relationship between the functional requirements and design parameters of the self-balancing two-wheeled vehicle in the design scheme is established, as shown in Table 8. The design matrix, as shown in Eq. (5), indicates a non-coupled design according to the definition of the AD theory, suggesting that the design scheme is theoretically reasonable.

**Table 8. The new mapping relationship between Functional Requirements and Design Parameters**

| **Functional Requirements (FRs)** | **Design Parameters (**$\boldsymbol{DP}_{\boldsymbol{s}}^{\boldsymbol{'}}$**)** |
| --- | --- |
| Ease of standing on the vehicle(FR_11_) | Footboard area ($\mathrm{DP}_{11}^{'}$) |
| Riding speed(FR_12_) | Hub motors and wheels($\mathrm{DP}_{12}^{'}$) |
| Stable structures(FR_21_) | Self-locking structure ($\mathrm{DP}_{21}^{'}$) |
| Responsive braking system(FR_22_) | Gyroscope and controller ($\mathrm{DP}_{22}^{'}$) |
| Good shock absorption (FR_31_) | Suspension structure ($\mathrm{DP}_{31}^{'}$) |
| Lightweight(FR_41_) | Materials with high strength and low density ($\mathrm{DP}_{41}^{'}$) |
| Handle for carrying(FR_42_) | T handle structure($\mathrm{DP}_{42}^{'}$) |
| Reduced size when idle (FR_43_) | Rotating shaft($\mathrm{DP}_{43}^{'}$) |
| Simple structures for easy maintenance (FR_51_) | Car frame ($\mathrm{DP}_{51}^{'}$) |
| Aesthetically pleasing appearance (FR_61_) | Appearance ($\mathrm{DP}_{61}^{'}$) |

$$\left\{ \begin{aligned} {FR}_{11} \\ {FR}_{12} \\ {FR}_{21} \\ {FR}_{22} \\ {FR}_{31} \\ {FR}_{41} \\ {FR}_{42} \\ {FR}_{43} \\ {FR}_{51} \\ {FR}_{61} \end{aligned} \right\}=\left[ \begin{aligned} X O O O O O O O O O \\ O X O O O O O O O O \\ O O X O O O O O O O \\ O O O X O O O O O O \\ O O O O X O O O O O \\ O O O O O X O O O O \\ O O O O O O X O O O \\ O O O O O O O X O O \\ O O O O O O O O X O \\ O O O O O O O O O X \end{aligned} \right]\cdot\left\{ \begin{aligned} {DP}_{11}^{'} \\ {DP}_{12}^{'} \\ {DP}_{21}^{'} \\ {DP}_{22}^{'} \\ {DP}_{31}^{'} \\ {DP}_{41}^{'} \\ {DP}_{42}^{'} \\ {DP}_{43}^{'} \\ {DP}_{51}^{'} \\ {DP}_{61}^{'} \end{aligned} \right\}$$

(5)

~~Secondly, based on the mechanical structure in the new design scheme, refine the design scheme.~~ Secondly, refine the design scheme based on the mechanical structure in the new design scheme. The design schematics of the self-balancing two-wheeled vehicle are shown in Fig 8. This self-balancing two-wheeled is different from traditional ones. It can serve as a means of transportation when the user needs to travel from one place to another, ~~but~~ and can also be folded by the user when not in use. The folding steps are shown in Fig 9. Taking into account both commuting and portability in terms of ergonomics, the product size is determined as depicted in Fig 10. The product has a simple and compact appearance, with colors mainly black and gray. And blue vehicle lights are used to enhance its technological sense. The main material options for the product body include aluminum alloy, magnesium alloy, or carbon fiber, which ensure strength while maintaining lightweight quality. These materials possess high plasticity and mature manufacturing processes, including sheet metal processing, ~~metal~~ spinning, ~~metal~~ stamping, deep drawing, and superplastic forming. In addition, the surface can be changed with spray painting or powder coating to increase corrosion resistance and scratch resistance.

**Fig 8. The design schematics of the portable self-balancing two-wheeled vehicle. (a) This figure shows the deployment state of a portable two-wheeled balancing vehicle. (b). This figure shows the folding state of a portable two-wheeled balancing vehicle.**

**Fig 9. The operation procedures of folding the self-balancing two-wheeled vehicle**

**Fig 10. Product size chart. (a) This figure shows the product size of the self-balancing two-wheeled vehicle as it is deployed. (b) This figure shows the product size of the self-balancing two-wheeled vehicle as it is folded.**

**Evaluation of the self-balancing two-wheeled vehicle design scheme**

First, the user requirements obtained through Kano analysis are transformed into evaluation system indicators, namely, commuting functionality(A), safety(B), comfort(C), portability(D), maintenance complexity(E), and aesthetics(F).

Next, divide the evaluation levels of each indicator and determine the design scope on user requirements. A-D and F are divided into five evaluation levels: poor, fair, average, good, and excellent, while E is divided into three evaluation levels: simple, average, and complex. The design ranges of each indicator are determined based on user requirements. The design range for A-D and F is good and above, while the design range for E is average and below.

Then, through expert evaluation, the two self-balancing two-wheeled vehicles are assessed, and the evaluation results are shown in Table 9.

**Table 9. The evaluation results of two self-balancing two-wheeled vehicles**

|  | **Commuting functionality (A)** | **Safety (B)** | **Comfort (C)** | **Portability (D)** | **Maintenance complexity (E)** | **Aesthetics (F)** |
| --- | --- | --- | --- | --- | --- | --- |
| **The design scheme** | Good | Good | Good | Excellent | Average | Good |
| **The original vehicle** | Excellent | Good | Good | Average | Simple | Average |

Finally, calculate the information amount based on the plotted function graph. Take the commuting function (A) as an example; the plotted function graph is shown in Fig 11. Eq. (6) and Eq. (7) show the information calculation processes.

**Fig 11. The membership function curve of the commuting function(A)**

$$I_{A\left( the design scheme \right)}={log}_{2}\left( \frac{fuzzy design scope}{fuzzy public scope} \right)={log}_{2}\left[ \frac{\left( 3+2 \right)\times1\times\frac{1}{2}}{\left( 3-1 \right)\times1\times\frac{1}{2}} \right]=1.3219$$

(6)

$$I_{A\left( the original vehicle \right)}={log}_{2}\left( \frac{fuzzy design scope}{fuzzy public scope} \right)={log}_{2}\left[ \frac{\left( 3+2 \right)\times1\times\frac{1}{2}}{\left( 3+1 \right)\times1\times\frac{1}{2}} \right]=0.3219$$

(7)

The information calculation results for all indicator parameters are shown in Table 10. The calculation results show that the total information content of the design proposal is lower than that of the original vehicle, indicating that the design proposal is superior according to the information axiom. By comparing individual evaluation indicators, it is evident that the design proposal excels primarily in improved portability.

**Table 10. Calculation results of information quantity for each evaluation indicator**

| **Evaluation indicators** | **The design scheme** | **The original vehicle** |
| --- | --- | --- |
| Commuting functionality(A) | 1.3219 | 0.3219 |
| Safety(B) | 1.3219 | 1.3219 |
| Comfort(C) | 1.3219 | 1.3219 |
| Portability(D) | 0.3219 | 3.3219 |
| Maintenance complexity(E) | 1.3219 | 0.3219 |
| Aesthetics (F) | 1.3219 | 3.3219 |
| Total | 6.9314 | 9.9314 |

**Response 2:**

**The original comments:**

*Discussion of the findings was quite weak. The authors should provide a clear and insightful discussion that explains how the study presents a new perspective on the theory and practices.*

*There is no conceptual comparison with existing approaches, no discussion on the benefits and drawbacks of the new approach. Thus discussions and comparative analyses should be added, also it is important to compare your method with literature ones.*

*Please elaborate on managerial insights from the industrial cases in a distinct section. Please mention them in the abstract and conclusion sections as well.*

**The summary of the comments:**

First, the introduction section needs to state the contributions and significance of the study clearly. Additionally, the discussion section should compare the advantages and limitations of the research through a literature review. Lastly, the discussion section should provide insights from a managerial perspective, and these managerial insights should also be mentioned in the abstract and conclusion sections.

**The authors’ answer：**

The summary of the key modifications made：

As the research novelty is redefined, the abstract, discussion, and conclusion sections have all been rewritten.

Firstly, the discussion section has been restructured. The revised discussion section consists of "Contribution, Significance, and Limitations" and "Insights from Managerial Perspectives." In the "Contribution, Significance, and Limitations" subsection, we explain the significance of the study, outline its strengths based on literature research, and discuss and summarize the limitations regarding the literature. In the "Insights from Managerial Perspectives" subsection, we discuss the possibility of optimizing the research methods from a managerial standpoint, incorporating literature and industry cases to illustrate our points.

Next, the conclusion section has been rewritten. The new conclusion succinctly summarizes the research content and methods, emphasizing the research's significance. Simultaneously, future research directions are proposed to address the limitations, referencing management studies.

Additionally, the abstract has been rewritten. The revised abstract concisely introduces the research background, methods, and significance, and mentions the managerial insights.

Last but not least, we have optimized the manuscript by improving the clarity of expression while modifying the content.

To facilitate the reviewer and prevent him from continuously switching between the "Response to Reviewers" and "Revised Manuscript with Track Changes" files, the following modifications will be presented comprehensively and specifically, following the sequence of abstract, discussion, and conclusion sections.

The specific changes and additions：

**Abstract**

~~In the era of rapid scientific and technological development, market competition has become increasingly fierce. In order to maintain their advantages, every industry considers rapid product iteration to be crucial. To enhance innovation capability, it is important to identify user needs and analyze and solve problems effectively. Axiomatic Design(AD), as a systematic theoretical approach, provides assistance to designers in analyzing design problems quantitatively. However, AD theory has limitations in identifying user needs and solving design problems. Kano, as a quantitative method, can help determine user needs and classify them into different levels. The Theory of the Solution of Inventive Problems(TRIZ) provides various tools to solve design problems. By combining these two methods with AD theory, the shortcomings of AD theory can be effectively addressed. This paper proposes a new product innovation design method that integrates Kano, TRIZ, and AD, this not only addresses the shortcomings of the AD theory but also ensures the completeness of the product design process, making it more applicable to practical product projects. The method consists of three main steps: firstly, using Kano to clarify user needs and their levels, and decomposing the functional requirements of the product based on user needs; secondly, utilizing the independence axiom in AD theory to establish the mapping relationship between functional requirements and design parameters, and analyze design problems; thirdly, applying contradiction analysis from TRIZ theory to solve problems. The feasibility and effectiveness of the method will be demonstrated by designing a self-balancing two-wheeled vehicle.~~

In the era of rapid product iteration, companies need simple and effective methods to guide the entire process of product innovation design and enhance their product innovation capabilities. Most research focused on improving one or several steps in the product design process. Although some scholars have proposed methods that guided the entire process, they combined more than three different theories, which increased the difficulty of theoretical learning and the complexity of practical implementation. This paper proposed a product innovation design process composed of three theoretical methods: Kano, Axiomatic Design (AD), and Theory of the Solution of Inventive Problems (TRIZ). This new process guided the entire product design process with fewer theoretical methods, reducing the difficulty of learning and implementation. The paper demonstrated the effectiveness of this method through the design practice of a portable two-wheeled self-balancing vehicle. Additionally, the discussion section explored the method's potential from the design management perspective.

**Discussion**

~~To further evaluate the design proposal, the information content of the product is calculated using the information axiom of the AD theory. A comparison is then made between the design scheme and the self-balancing two-wheeled vehicle before the design improvement.~~

~~The user requirements obtained through Kano analysis are transformed into evaluation system indicators, namely, commuting functionality(A), safety(B), comfort(C), portability(D), maintenance complexity(E), and aesthetics(F). Among them, A-D and F are divided into five evaluation levels: poor, fair, average, good, and excellent, while E is divided into three evaluation levels: simple, average, and complex. The design ranges of each indicator are determined based on user requirements. The design range for A-D and F is good and above, while the design range for E is average and below. Through expert evaluation, the two self-balancing two-wheeled vehicles are assessed, and the evaluation results are shown in Table 7.~~

**~~Table 7. The evaluation results of two self-balancing two-wheeled vehicles~~**

|  | **~~Commuting functionality (A)~~** | **~~Safety (B)~~** | **~~Comfort (C)~~** | **~~Portability (D)~~** | **~~Maintenance complexity (E)~~** | **~~Aesthetics (F)~~** |
| --- | --- | --- | --- | --- | --- | --- |
| **~~The design scheme~~** | ~~Good~~ | ~~Good~~ | ~~Good~~ | ~~Excellent~~ | ~~Average~~ | ~~Good~~ |
| **~~The original vehicle~~** | ~~Excellent~~ | ~~Good~~ | ~~Good~~ | ~~Average~~ | ~~Simple~~ | ~~Average~~ |

~~This paper adopts the triangular fuzzy function as the membership function to transform linguistic terms into fuzzy numbers [44]. Taking the commuting function(A) as an example, the membership function curve of the commuting function(A) is obtained based on the triangular fuzzy function, as shown in Fig 11.~~

**~~Fig 11. The membership function curve of the commuting function(A)~~**

~~Based on the formulas, the information content of the commuting functionality (A) for the two self-balancing electric scooters can be calculated as shown in Eq. (5) and Eq. (6). The calculation results of the information content for all indicator parameters are shown in Table 8.~~

|  | $I_{A\left( the design scheme \right)}={log}_{2}\left( \frac{fuzzy design scope}{fuzzy public scope} \right)={log}_{2}\left[ \frac{\left( 3+2 \right)\times1\times\frac{1}{2}}{\left( 3-1 \right)\times1\times\frac{1}{2}} \right]=1.3219$ | ~~（5）~~ |
| --- | --- | --- |
|  | $I_{A\left( the original vehicle \right)}={log}_{2}\left( \frac{fuzzy design scope}{fuzzy public scope} \right)={log}_{2}\left[ \frac{\left( 3+2 \right)\times1\times\frac{1}{2}}{\left( 3+1 \right)\times1\times\frac{1}{2}} \right]=0.3219$ | ~~（6）~~ |

**~~Table 8. Calculation results of information quantity for each evaluation indicator~~**

| **~~Evaluation indicators~~** | **~~The design scheme~~** | **~~The original vehicle~~** |
| --- | --- | --- |
| **~~Commuting functionality(A)~~** | **~~1.3219~~** | **~~0.3219~~** |
| **~~Safety(B)~~** | **~~1.3219~~** | **~~1.3219~~** |
| **~~Comfort(C)~~** | **~~1.3219~~** | **~~1.3219~~** |
| **~~Portability(D)~~** | **~~0.3219~~** | **~~3.3219~~** |
| **~~Maintenance complexity(E)~~** | **~~1.3219~~** | **~~0.3219~~** |
| **~~Aesthetics (F)~~** | **~~1.3219~~** | **~~3.3219~~** |
| **~~Total~~** | **~~6.9314~~** | **~~9.9314~~** |

~~From the calculation results, it can be observed that the total information content of the design proposal is lower than that of the original vehicle, indicating that the design proposal is superior according to the information axiom of the AD theory. By comparing individual evaluation indicators, it is evident that the design proposal excels primarily in terms of improved portability. Other optimization designs for self-balancing two-wheeled vehicles could consider making breakthroughs in terms of portability based on this observation.~~

**Contribution, Significance, and Limitations**

This paper proposed a process for product innovation design by combining Kano, AD, and TRIZ. The new process was applied to the design practice of a self-balancing two-wheeled vehicle, which resulted in an improved design scheme. The final design output demonstrated that the proposed method could effectively guide the entire product innovation design process, helping designers understand user needs, analyze functional requirements, identify and resolve structural contradictions, and evaluate design solutions. In the new method, Kano's identification of user needs clarifies the design direction in the early stages and forms an evaluation model for design solutions in conjunction with AD during the design evaluation stage. The AD theory plays a role in transforming user needs, analyzing design contradictions, and evaluating design solutions in the new process. These theoretical methods fully demonstrate their effectiveness and characteristics in the new process, leading to a reduced complexity of integrated methods in theory and practice compared to previous studies. [59,60].

However, shortcomings in the new process can also be clearly identified during design practice. Firstly, the method proposed in this paper cannot effectively identify dynamically changing user requirements. Reference [70] combined Kano, grey relational analysis, and benchmarking theory to help identify and calculate dynamically changing user requirements and satisfaction. Additionally, using TRIZ to find innovative methods can lengthen the design cycle. Although TRIZ has summarized many parameters and inventive principles, it still relies on the designer's experience for practical application selection. In this process, the design team needs to go through several trial-and-error attempts to find the correct direction, which undoubtedly prolongs the design cycle. Reference [71] proposed utilizing artificial neural networks (ANN) to address this issue. In conclusion, there are still shortcomings in the new process proposed in this study. Further research should try to address the shortcomings through optimization methods or by considering other perspectives.

**Insights from managerial perspectives**

Based on the above discussion, it can be concluded that the proposed method has limitations in both capturing dynamic user requirements and efficiently utilizing TRIZ. Appropriate management approaches can partially compensate for the drawbacks of new processes without increasing the complexity of theoretical methods in practical implementation. The following will explain the design management approach of agile development and discuss the potential of using this management approach to address the drawbacks based on literature research and successful design cases.

Agile development originally referred to a design and management approach for software development that emphasizes iteration and incremental progress [72]. It underlines rapid feedback, flexibility, and collaboration, focusing on team communication and cooperation to adapt to changing requirements and rapidly iterate product updates [73]. This design management approach has gradually been extended to physical product design, known as agile product development [74-76].

The agile product development management approach requires constant feedback from users during the product design process to adapt to user needs and adjust design plans promptly. Simultaneously, technical development for implementing product functionalities should be conducted while conceptualizing design plans, with regular feedback provided [77]. The application of the agile product development management approach in industrial case studies, as demonstrated in the literature [77-79], has proven the effectiveness of this management approach in responding to dynamic user needs and rapidly iterating product technology plans. Many commercially successful products have been developed using this design management approach, such as Tesla's Model 3. Tesla's design team closely collaborated with potential users during the design process, rapidly collecting feedback on requirements and opinions on automotive interior design and promptly adjusting design plans. Moreover, due to the parallel design and development management approach, Tesla could proceed with the release and sales of Model 3 without any delays, even in the event of design plan adjustments.

In summary, the agile product development management approach facilitates tracking changes in user requirements and rapid iteration of product solutions. This effectively compensates for the shortcomings of the proposed method process in this paper. It provides a research direction for the subsequent optimization of the new approach.

**Conclusion**

~~The design process proposed in this paper, with AD as the core, combines Kano and TRIZ, which effectively addresses the shortcomings of the AD in providing clear user requirements and lacking methods to assist in solving design problems. However, there are still limitations in this research. First, when dealing with complex mechanical products, errors may occur in functional modeling, leading to subsequent issues in the work. Second, when using the Achshuler contradiction matrix to find inventive principles, multiple inventive principles are often identified. The selection of inventive principles still requires a trial-and-error approach, which inevitably prolongs the design cycle. Future research will focus on two directions: improving the accuracy of functional modeling and proposing methods to assist in screening inventive principles to reduce the number of trial-and-error attempts.~~

The proposed design method combines Kano, AD, and TRIZ, covering the processes and steps of researching user needs, transforming needs into functional requirements, analyzing design problems, solving design problems, and evaluating design solutions. It guided the entire design process with fewer theoretical approaches, reducing the complexity of theory and practice. The design practice of a self-balancing two-wheeled vehicle demonstrated the new method's effectiveness. However, there are still limitations and shortcomings in this study. Firstly, the proposed theoretical method weakens the ability to capture dynamically changing user needs. Additionally, selecting appropriate engineering parameters and inventive principles from TRIZ relies on personal experience and multiple trial and error. From a management perspective, when designing practice according to the new process, combining the agile product development management approach may compensate for the new process's shortcomings without increasing theoretical complexity. Future research will attempt to optimize the proposed method process from the perspectives of management and other disciplines, enhancing the ability to follow up on changes in user needs and improving the accuracy of selecting engineering parameters and inventive principles.

**Reference:**

[1] Goli A., Tirkolaee E. B., and Aydın N. S. 2021. "Fuzzy Integrated Cell Formation and Production Scheduling Considering Automated Guided Vehicles and Human Factors." *IEEE Transactions on Fuzzy Systems* 29 (12):3686-3695. doi: <https://doi.org/10.1109/TFUZZ.2021.3053838>.

[2] Akgül Esra, Özmen Mihrimah, Sinanoğlu Cem, and Kizilkaya Aydoğan Emel. 2020. "Rough Kansei Mining Model for Market-Oriented Product Design." *Mathematical Problems in Engineering* 2020:6267031. doi: <https://doi.org/10.1155/2020/6267031>.

[3] Zou Fanxing, Zhu Xinyu, Qian Yuning, and Chang Danni. 2022. "A Sustainable Product Design Approach Based on Data Mining of Dynamic User Demands: A Case Study on HUAWEI Mate 40 Series." *IFAC-PapersOnLine* 55 (10):1056-1061. doi: <https://doi.org/10.1016/j.ifacol.2022.09.529>.

[4] Jin Hui Ying, Guo Haixia, Cao Guozhong, Wei Saina %J Applied Mechanics, and Materials. 2013. "Function Innovation Design Model and its Application in Heating Equipment." *Applied Mechanics and Materials* 401-403:371 - 374. doi: <https://doi.org/10.4028/www.scientific.net/AMM.401-403.371>.

[5] Gero John S., and Kannengiesser Udo. 2004. "The situated function–behaviour–structure framework." *Design Studies* 25 (4):373-391. doi: <https://doi.org/10.1016/j.destud.2003.10.010>.

[6] Zhiguo Zhang. 2009. "Research on product design based on the Axiomatic design." 2009 IEEE 10th International Conference on Computer-Aided Industrial Design & Conceptual Design, 26-29 Nov. 2009.

[7] Kano Noriaki, Seraku Nobuhiko, Takahashi Fumio, and Tsuji Shin-ichi %J JOURNAL OF THE JAPANESE SOCIETY FOR QUALITY CONTROL. 1984. "Attractive Quality and Must-Be Quality." *Journal of Japanese Society for Quality Control* 14:39-48.

[8] Lee Ching-Hung, Chen Chun-Hsien, Lin Chenyu, Li Fan, and Zhao Xuejiao. 2019. "Developing a Quick Response Product Configuration System under Industry 4.0 Based on Customer Requirement Modelling and Optimization Method." *Applied Sciences* 9 (23):5004. doi: <https://doi.org/10.3390/app9235004>.

[9] Chen Yung-Hsin, and Su Chao-Ton. 2006. "A Kano-CKM model for customer knowledge discovery." *Total Quality Management & Business Excellence* 17 (5):589-608. doi: <https://doi.org/10.1080/14783360600588158>.

[10] Kuo Ying-Feng. 2004. "Integrating Kano’s Model into Web- community Service Quality." *Total Quality Management & Business Excellence* 15 (7):925-939. doi: <https://doi.org/10.1080/14783360410001681854>.

[11] Tontini Gérson. 2000. "Identification of customer attractive and must-be requirements using a modified Kano's method: guidelines and case study." ASQ's 54th Annual Quality Congress Proceedings, 01/01.

[12] Kano N., and Takahashi F. 1979. "On M-H property of quality." Nippon QC Gakkai, 01/01.

[13] Violante Maria Grazia, and Vezzetti Enrico. 2017. "Kano qualitative vs quantitative approaches: An assessment framework for products attributes analysis." *Computers in Industry* 86:15-25. doi: <https://doi.org/10.1016/j.compind.2016.12.007>.

[14] Suh N. P. 1995. "Axiomatic Design of Mechanical Systems." *Journal of Vibration and Acoustics* 117 (B):2-10. doi: <https://doi.org/10.1115/1.2838673>.

[15] Lee Taesik. 2003. "Complexity theory in axiomatic design." Doctor, the Department of Mechanical Engineering, Massachusetts Institute of Technology.

[16] Farid Amro M., and Suh Nam P. 2016. *Axiomatic Design in Large Systems*. Cham, Switzerland: Springer International Publishing AG Switzerland.

[17] Suh Nam Pyo. 2001. *Axiomatic Design: Advances and Applications*. New York, USA: Oxford University Press.

[18] Shirwaiker Rohan A., and Okudan Gül E. 2008. "Triz and axiomatic design: a review of case-studies and a proposed synergistic use." *Journal of Intelligent Manufacturing* 19 (1):33-47. doi: <https://doi.org/10.1007/s10845-007-0044-6>.

[19] Deng Xinyang, and Jiang Wen. 2018. "An Evidential Axiomatic Design Approach for Decision Making Using the Evaluation of Belief Structure Satisfaction to Uncertain Target Values." *International Journal of Intelligent Systems* 33 (1):15-32. doi: <https://doi.org/10.1002/int.21929>.

[20] Simon Herbert A. 1996. *The sciences of the artificial (3rd ed.)*. Cambridge, MA, USA: MIT Press.

[21] Park Sang-ok, Yoon Jongmin, An Hochan, Park Jeonggyu, and Park Gyung-Jin. 2021. "Integration of axiomatic design and design structure matrix for the modular design of automobile parts." *Proceedings of the Institution of Mechanical Engineers, Part B: Journal of Engineering Manufacture* 236 (3):296-306. doi: <https://doi.org/10.1177/09544054211014484>.

[22] Wu Xinping, Geng Xinzhou, Chen Zhiyi, Dong Aidi, and Li Jinchao. 2022. "Dynamic Design Quality Evaluation of Power Enterprise Digital System Based on Fuzzy Information Axiom." *Mathematical Problems in Engineering* 2022:2617640. doi: <https://doi.org/10.1155/2022/2617640>.

[23] Kumar Prabhat, and Tandon Puneet. 2019. "A paradigm for customer-driven product design approach using extended axiomatic design." *Journal of Intelligent Manufacturing* 30 (2):589-603. doi: <https://doi.org/10.1007/s10845-016-1266-2>.

[24] Shirwaiker R. A., and Okudan G. E. 2006. "TRIZ and Axiomatic Design: A Review of Manufacturing Case-Studies & Their Compatibility." 2006 Technology Management for the Global Future - PICMET 2006 Conference, 8-13 July 2006.

[25] Chang Yu-Shan, Chien Yu-Hung, Yu Kuang-Chao, Chu Yih-Hsien, and Chen Mavis Yi-ching. 2016. "Effect of TRIZ on the creativity of engineering students." *Thinking Skills and Creativity* 19:112-122. doi: <https://doi.org/10.1016/j.tsc.2015.10.003>.

[26] Ruihong Zhang, Jianzhong Cha, and Yiping Lu. 2007. "A conceptual design model using axiomatic design, functional basis and TRIZ." 2007 IEEE International Conference on Industrial Engineering and Engineering Management, 2-4 Dec. 2007.

[27] Chou Jyh-Rong. 2021. "A TRIZ-based product-service design approach for developing innovative products." *Computers & Industrial Engineering* 161:107608. doi: <https://doi.org/10.1016/j.cie.2021.107608>.

[28] Jafari Mostafa, and Zarghami Hamid Reza. 2017. "Effect of TRIZ on enhancing employees’ creativity and innovation." *Aircraft Engineering and Aerospace Technology* 89 (6):853-861. doi: <https://doi.org/10.1108/AEAT-01-2016-0004>.

[29] Pin Soo Chin, Haron Fazilah, Sarmady Siamak, Talib Abdullah Zawawi, and Khader Ahamad Tajudin. 2011. "Applying TRIZ principles in crowd management." *Safety Science* 49 (2):286-291. doi: <https://doi.org/10.1016/j.ssci.2010.09.002>.

[30] Russo D., Rizzi C., and Montelisciani G. 2014. "Inventive guidelines for a TRIZ-based eco-design matrix." *Journal of Cleaner Production* 76:95-105. doi: <https://doi.org/10.1016/j.jclepro.2014.04.057>.

[31] Li Miao, Ming Xinguo, He Lina, Zheng Maokuan, and Xu Zhitao. 2015. "A TRIZ-based Trimming method for Patent design around." *Computer-Aided Design* 62:20-30. doi: <https://doi.org/10.1016/j.cad.2014.10.005>.

[32] Mann Darrell L. 2007. *Hands-on systematic innovation*. Frankfurt, Germany: IFR Press.

[33] Cheng Guang. 2015. *Innovative Thinking and Methods: Theory and Application of TRIZ*. China, Beijing: Science Press.(In Chinese)

[34] Dubois Sébastien, Guio Roland De, and Rasovska Ivana. 2011. "Resolution of inventive problems: different kind of mechanisms." *International Journal of Systematic Innovation* 1 (4):2-11.

[35] Sheu D. Daniel, Chiu Ming-Chuan, and Cayard Dimitri. 2020. "The 7 pillars of TRIZ philosophies." *Computers & Industrial Engineering* 146:106572. doi: <https://doi.org/10.1016/j.cie.2020.106572>.

[36] Tan Runhua. 2010. *TRIZ and Applications*.China, Beijing: Higher Education Press. (In Chinese)

[37] Chen Jiwen, Li Xin, Yang Hongjuan, and Wang Chen. 2019. "Innovative Design of a Vertical and Transverse Elevator in Double Shafts, Based on TRIZ Theory." *Strojniški vestnik - Journal of Mechanical Engineering* 65 (5):14 doi: <https://doi.org/10.5545/sv-jme.2018.5937>.

[38] Almeida Sergio T., Mo John, Bil Cees, Ding Songlin, and Wang Xiangzhi. 2021. "Conceptual Design of a High-Speed Wire EDM Robotic End-Effector Based on a Systematic Review Followed by TRIZ." *Machines* 9 (7):132. doi: <https://doi.org/10.3390/machines9070132>.

[39] Huang Binda, Zhou Laishui, An Luling, Wei wei, and Bu Qingkui. 2017. " Axiomatic Design of TRIZ-integrated Machining Fifixture Scheme." *Chinese Journal of Scientific Instrument* 38 (04):1031-1040. doi: <https://doi.org/10.19650/j.cnki.cjsi.2017.04.030>. (In Chinese)

[40] Hosseinpour Arash, and Peng Qingjin. 2012. "Sustainable Design Using Integrated TRIZ and Eco-Checklist With Function Impact Matrix."

[41] Butdee Suthep, and Vignat Frédéric. 2008. "TRIZ method for light weight bus body structure design." *Journal of Achievements in Materials and Manufacturing Engineering* 31 (2):456-462.

[42] Du Laihong, Chen Hua, Fang Yadong, Liang Xiaowei, Zhang Yujie, Qiao Yidan, and Guo Zhen. 2022. "Research on the Method of Acquiring Customer Individual Demand Based on the Quantitative Kano Model." *Computational Intelligence and Neuroscience* 2022:5052711. doi: <https://doi.org/10.1155/2022/5052711>.

[43] Goli Alireza, Khademi Zare Hasan, Tavakkoli-Moghaddam Reza, and Sadeghieh Ahmad. 2019. "Hybrid artificial intelligence and robust optimization for a multi-objective product portfolio problem Case study: The dairy products industry." *Computers & Industrial Engineering* 137:106090. doi: <https://doi.org/10.1016/j.cie.2019.106090>.

[44] Goli Alireza, Khademi-Zare Hasan, Tavakkoli-Moghaddam Reza, Sadeghieh Ahmad, Sasanian Mazyar, and Malekalipour Kordestanizadeh Ramina. 2021. "An integrated approach based on artificial intelligence and novel meta-heuristic algorithms to predict demand for dairy products: a case study." *Network: Computation in Neural Systems* 32 (1):1-35. doi: <https://doi.org/10.1080/0954898X.2020.1849841>.

[45] Kim Taehoon, Lim Hyunsu, and Cho Kyuman. 2022. "Conceptual robot design for the automated layout of building structures by integrating QFD and TRIZ." *The International Journal of Advanced Manufacturing Technology* 120 (3):1793-1804. doi: <https://doi.org/10.1007/s00170-022-08803-2>.

[46] Yang Mei, Xia Yimin, Jia Lianhui, Wang Dujuan, and Ji Zhiyong. 2021. "A modular design method based on TRIZ and AD and its application to cutter changing robot." *Advances in Mechanical Engineering* 13 (7):1-18. doi: <https://doi.org/10.1177/16878140211034369>.

[47] Li Xiuzhen, Qiu Siqi, and Ming Henry X. G. 2019. "An integrated module-based reasoning and axiomatic design approach for new product design under incomplete information environment." *Computers & Industrial Engineering* 127:63-73. doi: <https://doi.org/10.1016/j.cie.2018.11.057>.

[48] Bai Zhong-hang, Zhang Shan, Ding Man, and Sun Jian-guang. 2018. "Research on product innovation design of modularization based on theory of TRIZ and axiomatic design." *Advances in Mechanical Engineering* 10 (12):1687814018814087. doi: <https://doi.org/10.1177/1687814018814087>.

[49] Lyu Jianhua, Chen Ran, Yang Lingyun, Wang Jialei, and Chen Ming. 2022. Applying a Hybrid Kano/Quality Function Deployment Integration Approach to Wood Desk Designs for Open-Plan Offices. *Forests* 13 (11). doi:<https://doi.org/10.3390/f13111825>.

[50] Zhu J., and Hou H. 2021. "Research on User Experience Evaluation of Mobile Applications in Government Services." *IEEE Access* 9:52634-52641. doi: <https://doi.org/10.1109/ACCESS.2021.3070365>.

[51] Tandiono Yauwseph, and Rau Hsin. 2023. An Enhanced Model Using the Kano Model, QFDE, and TRIZ with a Component-Based Approach for Sustainable and Innovative Product Design. *Sustainability* 15 (1). doi:<https://doi.org/10.3390/su15010527>.

[52] Yao Kai-Chao, Huang Wei-Tzer, Xu Jing-Ran, Huang Shu-Hua, Tsai Chin-Tang, Ho Wei-Sho, and Liao Chun-Chung. 2023. Application of the TRIZ Innovation System Method to Bicycle Handlebars. *Machines* 11 (5). doi:<https://doi.org/10.3390/machines11050507>.

[53] Zhu Tian-Lu, Li Ya-Jun, Wu Ceng-Juan, Yue Han, and Zhao Yi-Qian. 2022. "Research on the Design of Surgical Auxiliary Equipment Based on AHP, QFD, and PUGH Decision Matrix." *Mathematical Problems in Engineering* 2022:4327390. doi: <https://doi.org/10.1155/2022/4327390>.

[54] Zhang Xin, Li Jiehao, Fan Ke, Chen Ziyang, Hu Zhenhuan, and Yu Yu. 2020. Neural Approximation Enhanced Predictive Tracking Control of a Novel Designed Four-Wheeled Rollator. *Applied Sciences* 10 (1). doi:<https://doi.org/10.3390/app10010125>.

[55] Li Yongchuan, Ghazilla Raja A., and Abdul-Rashid Salwa H. 2022. QFD-Based Research on Sustainable User Experience Optimization Design of Smart Home Products for the Elderly: A Case Study of Smart Refrigerators. *International Journal of Environmental Research and Public Health* 19 (21). doi:<https://doi.org/10.3390/ijerph192113742>.

[56] Wang Nanyi, Shi Chang, and Kang Xinhui. 2022. Design of a Disinfection and Epidemic Prevention Robot Based on Fuzzy QFD and the ARIZ Algorithm. *Sustainability* 14 (24). doi:<https://doi.org/10.3390/su142416341>.

[57] Wang Tianxiong, and Yang Liu. 2023. Combining GRA with a Fuzzy QFD Model for the New Product Design and Development of Wickerwork Lamps. *Sustainability* 15 (5). doi:<https://doi.org/10.3390/su15054208>.

[58] Moran Daniel, Ertas Atila, and Gulbulak Utku. 2021. A Unique Transdisciplinary Engineering-Based Integrated Approach for the Design of Temporary Refugee Housing Using Kano, HOQ/QFD, TRIZ, AD, ISM and DSM Tools. *Designs* 5 (2). doi:<https://doi.org/10.3390/designs5020031>.

[59] Zhao Deng, and Chen Weiwei. 2021. "Design and Research of Smart Neck Helmets Based on the KANO-QFD Model and TRIZ Theory." *Security and Communication Networks* 2021:4693719. doi: <https://doi.org/10.1155/2021/4693719>.

[60] Hameed Abdul Z., Kandasamy Jayakrishna, Aravind Raj Sakthivel, Baghdadi Majed A., and Shahzad Muhammad A. 2022. Sustainable Product Development Using FMEA ECQFD TRIZ and Fuzzy TOPSIS. *Sustainability* 14 (21). doi:<https://doi.org/10.3390/su142114345>.

[61] Wang Jian-wei, and Zhang Jian-min. 2019. "Research on Innovative Design and Evaluation of Agricultural Machinery Products." *Mathematical Problems in Engineering* 2019:8179851. doi: <https://doi.org/10.1155/2019/8179851>.

[62] Neira-Rodado Dionicio, Ortíz-Barrios Miguel, De la Hoz-Escorcia Sandra, Paggetti Cristiano, Noffrini Laura, and Fratea Nicola. 2020. Smart Product Design Process through the Implementation of a Fuzzy Kano-AHP-DEMATEL-QFD Approach. *Applied Sciences* 10 (5). doi:<https://doi.org/10.3390/app10051792>.

[63] Fang Meichen, Yang Wei, Li Hui, and Pan Younghwan. 2023. Enhancing User Experience through Optimization Design Method for Elderly Medication Reminder Mobile Applications: A QFD-Based Research Approach. *Electronics* 12 (13). doi:<https://doi.org/10.3390/electronics12132860>.

[64] Zhang Baoyi, Ma Minyuan, and Wang Zongsheng. 2023. "Promoting active aging through assistive product design innovation: a preference-based integrated design framework." 11. doi: <https://doi.org/10.3389/fpubh.2023.1203830>.

[65] Wu Xiaoli, Hong Zhuang, Li Yajun, Zhou Feng, Niu Yafeng, and Xue Chengqi. 2020. "A function combined baby stroller design method developed by fusing Kano, QFD and FAST methodologies." *International Journal of Industrial Ergonomics* 75:102867. doi: <https://doi.org/10.1016/j.ergon.2019.102867>.

[66] Wu Chunlong, Zhou Youcheng, Pereia Pessôa Marcus Vinicius, Peng Qingjin, and Tan Runhua. 2021. "Conceptual digital twin modeling based on an integrated five-dimensional framework and TRIZ function model." *Journal of Manufacturing Systems* 58:79-93. doi: <https://doi.org/10.1016/j.jmsy.2020.07.006>.

[67] Liang Kai, Yang Jie, Long Xiaobin, Liang Xuemei, Lu Jia, and Liu Xiong. 2020. " Avoidance and Innovation Design of Multilateral Bending Machine Based on Functional Modeling." *Journal of Guangdong University of Technology* 37 (01):7-14.(In Chinese)

[68] Xu Xinzhao, and Fang Jun. 2019. " Research on the Evaluation and Application of the Configurable Product Design Scheme Based on the Axiom of Fuzzy Information." *JOURNAL OF MACHNIE DESIGN* 36 (07):14-20. doi: <https://doi.org/10.13841/j.cnki.jxsj.2019.07.003>.(In Chinese)

[69] Ai Dong, Li Jiangui, Liu Yanbo, and Luo Ruiren. 2021. " Design of New Integrated Permanent-magnet Hub Motor and Cycloidal Reducer System for Electrical Balanced Unicycle." (09):75-80. doi: <https://doi.org/10.19356/j.cnki.1001-3997.2021.09.018>.(In Chinese)

[70] Dou Runliang, Li Wei, and Nan Guofang. 2019. "An integrated approach for dynamic customer requirement identification for product development." *Enterprise Information Systems* 13 (4):448-466. doi: <https://doi.org/10.1080/17517575.2018.1526321>.

[71] Dou Runliang, Zhang Yubo, and Nan Guofang. 2017. "Iterative product design through group opinion evolution." *International Journal of Production Research* 55 (13):3886-3905. doi: <https://doi.org/10.1080/00207543.2017.1316020>.

[72] Conforto Edivandro C., Salum Fabian, Amaral Daniel C., da Silva Sérgio Luis, and de Almeida Luís Fernando Magnanini. 2014. "Can Agile Project Management be Adopted by Industries Other than Software Development?" *Project Management Journal* 45 (3):21-34. doi: <https://doi.org/10.1002/pmj.21410>.

[73] Kettunen Petri, and Laanti Maarit. 2008. "Combining agile software projects and large-scale organizational agility." *Software Process: Improvement and Practice* 13 (2):183-193. doi: <https://doi.org/10.1002/spip.354>.

[74] Conforto Edivandro C., and Amaral Daniel C. 2016. "Agile project management and stage-gate model—A hybrid framework for technology-based companies." *Journal of Engineering and Technology Management* 40:1-14. doi: <https://doi.org/10.1016/j.jengtecman.2016.02.003>.

[75] Schuh G., Dölle C., Kantelberg J., and Menges A. 2018. "Identification of Agile Mechanisms of Action As Basis for Agile Product Development." *Procedia CIRP* 70:19-24. doi: <https://doi.org/10.1016/j.procir.2018.02.007>.

[76] de Borba João Carlos R., Trabasso Luís Gonzaga, and Pessôa Marcus Vinicius P. 2019. "Agile Management in Product Development." *Research-Technology Management* 62 (5):63-67. doi: <https://doi.org/10.1080/08956308.2019.1638488>.

[77] Shim Woogon, and Lee Seok-Won. 2019. "An agile approach for managing requirements change to improve learning and adaptability." *Journal of Industrial Information Integration* 14:16-23. doi: <https://doi.org/10.1016/j.jii.2018.07.005>.

[78] Cooper Robert G., and Sommer Anita F. 2016. "The Agile–Stage-Gate Hybrid Model: A Promising New Approach and a New Research Opportunity." *Journal of Product Innovation Management* 33 (5):513-526. doi: <https://doi.org/10.1111/jpim.12314>.

[79] Coimbra Henrique, Cormican Kathryn, McDermott Olivia, and Antony Jiju. 2023. "Leading the transformation: agile success factors in an Irish manufacturing company." *Total Quality Management & Business Excellence*:1-28. doi: <https://doi.org/10.1080/14783363.2023.2214515>.
